# Novel waves structures for the nonclassical Sobolev-type equation in unipolar semiconductor with its stability analysis

**DOI:** 10.1038/s41598-023-47838-7

**Published:** 2023-12-17

**Authors:** Tahir Shahzad, Muhammad Ozair Ahmed, Muhammad Zafarullah Baber, Nauman Ahmed, Ali Akgül, Sayed M. El Din

**Affiliations:** 1 Department of Mathematics and Statistics, The University of Lahore, Lahore, Pakistan; 2 Department of Basic Sciences and Humanities, Narowal Campus, University of Engineering and Technology, Lahore, 54890 Pakistan; 3 Department of Computer Science and Mathematics, Lebanese American University, Beirut, Lebanon; 4 Department of Mathematics, Art and Science Faculty, Siirt University, 56100 Siirt, Turkey; 5 Mathematics Research Center, Department of Mathematics, Near East University, Near East Boulevard, 99138 Nicosia /Mersin 10, Turkey; 6 Center of Research, Faculty of Engineering, Future University in Egypt, New Cairo, 11835 Egypt

**Keywords:** Applied mathematics, Pure mathematics

## Abstract

In this study, the Sobolev-type equation is considered analytically to investigate the solitary wave solutions. The Sobolev-type equations are found in a broad range of fields, such as ecology, fluid dynamics, soil mechanics, and thermodynamics. There are two novel techniques used to explore the solitary wave structures namely as; generalized Riccati equation mapping and modified auxiliary equation (MAE) methods. The different types of abundant families of solutions in the form of dark soliton, bright soliton, solitary wave solutions, mixed singular soliton, mixed dark-bright soliton, periodic wave, and mixed periodic solutions. The linearized stability of the model has been investigated. Solitons behave differently in different circumstances, and their behaviour can be better understood by building unique physical problems with particular boundary conditions (BCs) and starting conditions (ICs) based on accurate soliton solutions. So, the choice of unique physical problems from various solutions is also carried out. The 3D, line graphs and corresponding contours are drawn with the help of the Mathematica software that explains the physical behavior of the state variable. This information can help the researchers in their understanding of the physical conditions.

## Introduction

Various physical phenomena can be formulated by a set of equations that help to understand and predict future events. In the past twenty years, partial differential equations (PDEs) have been used to study a wide range of natural phenomena^1–5^. The flow of fluids with fissured rock, thermodynamics, finance, ecology, mathematical physics, soil mechanics, and heat conduction difficulties in diverse materials are some of the disciplines where nonlinear PDEs and nonlinear Sobolev equations arise. Nonlinear PDEs better describe physical processes^6–12^. PDE analytical solutions are a dynamic field of study. The PDEs’ solutions are obtained by applying a variety of analytical techniques. Gómez gave the Sobolev-type equations some thought. The many forms of solutions, including periodic and soliton solutions, were obtained by the author through the analytical investigation using the generalised Tanh-Coth technique^13^. Aristov worked on the Sobolev equations’ precise solutions. He acquired the many families of the quadrature and elementary function solutions^14^. For the analytical investigation of various mathematical physics of nonlinear equations, Polyanan et al. employed a modified version of functional separation of variables techniques. Implicit shape and extended travelling wave solutions were the obtained solutions^15^.

By using the Hirota bilinear approach to the geophysical Korteweg-de Varies problem, Rizvi et al. were able to get several types of solutions, including lump-periodic soliton, lump-kink soliton, and lump-kind periodic solutions^16^. The $G^{\prime } /G^{2}$ expansion method and the expansion function methodology were two unique techniques used by the authors to study the Gilson-Pickering equation. They succeeded in obtaining the answers in the form of singular, shock, shock singular, periodic, rational, and singular periodic waves (citation 17). Cheema et al. worked on the solutions of Maccari’s system and Generalized elliptic equation with the extended fan technique. The authors gained different types of solutions, like triangular-type solutions, soliton-like solutions, and single and combined non-degenerate Jacobi elliptic wave function-like solutions^18^. Lu et al. considered the unstable nonlinear Schrödinger equation and used the modified form of the simple equation methods to gain solutions of the various types, namely trigonometric, rational, hyperbolic, and exponential functions^19^. Arshad et al. discussed the higher ordered nonlinear schrödinger equations analytically with the NMEDA approach and obtained various trigonometric solutions^20^. Inc et al. worked on the solitary wave solution of the Sawada-Kotera equation with two different techniques and obtained numerous solutions^21^.

We are considering the nonclassical Sobolev equation such as

1 $$\frac{\partial }{\partial t}\vartriangle {\Psi } + \vartriangle {\Psi } - \mu \left| {\Psi } \right|^{q} = 0,$$

here $\Psi$ denotes the real value of the function of the spatial variable $x$ and $t>0$, $\mu$ is a real value except zero and $q>1$ is a natural number. It explains the quasi-stationary approaches that occur in a unipolar semiconductor when the free charge current of source is supply. Here $\vartriangle = \frac{{\partial^{2} }}{{\partial x^{2} }} + \frac{{\partial^{2} }}{{\partial y^{2} }}$ denotes the higher dimensional spatial variable, $q$ stands for the nonlinearity of the unknown function and we take $q = 4$ is used for this study. So, we can modify the nonclassical Sobolev Eq. (1) as given below,

2 $$\frac{\partial }{\partial t}\left( {\frac{{\partial^{2} {\Psi }}}{{\partial x^{2} }} + \frac{{\partial^{2} {\Psi }}}{{\partial y^{2} }}} \right) + \left( {\frac{{\partial^{2} {\Psi }}}{{\partial x^{2} }} + \frac{{\partial^{2} {\Psi }}}{{\partial y^{2} }}} \right) - \mu {\Psi }^{4} = 0,$$

When we talk about analytical solutions in mathematics, we usually mean solutions that are stated in terms of mathematical functions, such as infinite series or other precisely defined mathematical expressions. Both series and explicit (closed-form) representations are possible in analytical solutions. Usually, these answers are obtained by applying different mathematical operations to the differential equations. Solutions that satisfy a particular differential equation exactly, devoid of any approximation, are referred to as exact solutions. These solutions could be explicit formulas or analytical solutions, which are expressed in terms of mathematical functions or series. Particularly when it comes to partial differential equations, precise solutions are frequently interchangeable with analytical solutions.

Alquran, M., et al., Heart-cusp and bell-shaped-cusp optical solitons for the complex Hirota model^22^, and multiplicative of dual-waves dual-mode Schrödinger with nonlinearity Kerr laws^23^. Jaradat, et al., discussed the numerical solutions of weak-dissipative two-mode perturbed Burgers’ and Ostrovsky models^24^ and solitary two-wave solutions for a new two-mode version of the Zakharov-Kuznetsov equation^25^. He also constructed the variety of physical structures to the generalized equal-width equation derived from Wazwaz-Benjamin-Bona-Mahony model^26^ and the combination of Dark-Bright Binary-Soliton derived from the (2 + 1)-dimensional Nizhnik-Novikov-Veselov (TMNNV) equation^27^.

There are many techniques that are developed to gained the exact solitary wave solutions such as generalized exponential rational function method^28–32^, Hirota’s bilinear transform^33, 34^, Jacobian elliptic functions method^35^, and etc. Ghanbari, B. used the generalized exponential rational function method and constructed the different form of optical soliton solutions for the Hirota-Maccari equation^36^ and Kundu-Mukherjee-Naskar equation^37^, generalized Schamel equation [?]. Also, he explored the new exact wave solutions of the variable-coefficient (1 + 1)-dimensional Benjamin-Bona-Mahony and (2 + 1)-dimensional asymmetric Nizhnik-Novikov-Veselov equations are constructed^38^, and for the gardner’s equation^39^. The soliton solutions are investigated for the modified nonlinear Schrödinger equations^40^ and (4 + 1)-dimensional nonlinear evolution equation^41^.

In this study we used two techniques namely as New Auxiliary Equation (NAE) technique and the Generalised Riccati Equation (GRE) mapping method. Depending on your preferences and the particular problem you are attempting to address, you can choose between the New Auxiliary Equation (NAE) technique and the Generalised Riccati Equation (GRE) mapping method. Both approaches are instruments for solving specific kinds of differential equations, and the situation at hand will determine how successful they are. Nonlinear ordinary differential equations (ODEs) can be solved by the GRE mapping method, which converts them into Riccati differential equations. When used properly, this approach can be very effective. It is especially helpful for various applications related to control theory, optimum control issues, and specific kinds of mathematical modelling. In certain situations, the differential equations may become simpler as a result of the GRE approach, making them easier to analyse and solve numerically. This method have different types of 27 abundant solutions. The NAE approach is useful in many engineering and physics contexts because it works effectively in situations when the differential equation’s coefficients are constants. Second-order linear differential equations, such as those describing oscillatory and harmonic systems, can be solved well using this method. It might not be appropriate for handling time-varying coefficients or more complicated nonlinear differential equations. Moreover it have not verity of the solutions it contains only five types of solutions.

The Generalized Riccati Equation Mapping Method and the New Auxiliary Equation Mapping Method are both mathematical techniques used to find exact solitary wave solutions to various nonlinear partial differential equations (PDEs). Each method has its strengths and weaknesses. These methods can be applied to a wide range of nonlinear PDEs, making it versatile in solving various physical and mathematical problems. It provides a general framework for finding solitary wave solutions, which can be customized and adapted for specific PDEs. These methods can involve complex algebraic manipulations and may require specialized mathematical skills to apply effectively, especially for more intricate PDEs. Success in finding exact solutions depends on the specific PDE and its characteristics. There is no guarantee that it will work for all PDEs. The main innovations of the manuscript are that the non-classical Sobolev equation is under consideration analytically. The analytical solutions are carried out with two techniques. The stability of the model is also discussed. New families of the solutions are obtained. The unique physical problems are chosen from various solutions. Graphical behavior is represented for various solutions.

## Extraction of exact solutions

In this part, the specific solutions of Eq. (1) must be found, applying the transformation by converting PDE into ODE for ${\Psi }$ = $w\left( \eta \right)$, here $\eta = \zeta_{1} x + \zeta_{2} y + \zeta_{3} t$. Where $\zeta_{1}$, $\zeta_{2}$, $\zeta_{3}$ and $w$ are the constants and a actual value function respectively. Hence, by replacing the above modification into Eq. (1), we receive the ODE develop as given below

3 $$\zeta_{3} \left( {\zeta_{1}^{2} + \zeta_{2}^{2} } \right)w^{\prime\prime\prime}\left( \eta \right) + \left( {\zeta_{1}^{2} + \zeta_{2}^{2} } \right)w^{\prime\prime}\left( \eta \right) - \mu w^{4} \left( \eta \right) = 0,$$

where $w$ is a polynomial and ${^{\prime}} = \frac{d}{d\eta }$.

Also, we take the solution of Eq. (2) and get the polynomials develop as^43, 44^,

4 $$w\left( \eta \right) = \lambda_{0} + \mathop \sum \limits_{j = 1}^{M} \lambda_{i} \psi^{M} \left( \eta \right),$$

where the constants $$\lambda_{0}$$ and $$\lambda_{j}$$ (i = 1,2,3,…M) that can be solve to be later,here $$\psi (\eta$$$$)$$ is simplify the Reccati Eq. as given below.

5 $$\psi ^{\prime}\left( \eta \right) = \left( {b_{0} + b_{1} \psi \left( \eta \right) + b_{2} \psi (\eta )^{2} } \right).$$

Homogenous balancing principle can be applied to find the value of $K$ in the previous Eq. (3) and we can enter $M = 1$ in Eq. (4)

6 $$w\left( \eta \right) = \lambda_{0} + \lambda_{1} \psi \left( \eta \right).$$

Determine the derivatives of Eq. (6) by applying the Eq. (5) and replace in the Eq. (3). After simplifying, collect each coefficients of the identical power of $\psi$ and set them then all to zero to gain a equations of system. Apply mathematica to deal with the system of calculation and gain the family of solution as,

### Family of solution

$$\lambda_{0} = \frac{{5\sqrt[3]{{{\Omega }_{1}^{2} + {\Omega }_{2}^{2} }}}}{{8 6^{2/3} \sqrt[3]{\mu }{\Omega }_{3}^{2/3} }},\lambda_{1} = \frac{{\sqrt[3]{6}b_{3} \sqrt[3]{{{\Omega }_{1}^{2} + {\Omega }_{2}^{2} }}\sqrt[3]{{{\Omega }_{3} }}}}{{\sqrt[3]{\mu }}},b_{1} = \frac{25}{{2304b_{3} {\Omega }_{3}^{2} }},b_{2} = \frac{1}{{24{\Omega }_{3} }}.$$

**Type 1:** When $b_{2}^{2} - 4b_{3} b_{1} > 0$ and $b_{3} b_{1} \ne 0$, then the twelve type of hyperbolic solutions exist such as,

7 $$w_{1} \left( {x,y,t} \right) = \frac{{5\sqrt[3]{{{\Omega }_{1}^{2} + {\Omega }_{2}^{2} }}}}{{8 6^{2/3} \sqrt[3]{\mu }{\Omega }_{3}^{2/3} }} - \frac{{\left( {\sqrt[3]{3}\sqrt[3]{{{\Omega }_{1}^{2} + {\Omega }_{2}^{2} }}\sqrt[3]{{{\Omega }_{3} }}} \right)}}{{2^{2/3} \sqrt[3]{\mu }}}\left( {\frac{1}{{24{\Omega }_{3} }} + \frac{{{\text{tanh}}\left( {\frac{{\alpha_{1} x + \alpha_{2} y + \alpha_{3} t}}{{\sqrt { - 96{\Omega }_{3}^{2} } }}} \right)}}{{\sqrt { - 24{\Omega }_{3}^{2} } }}} \right),$$

8 $$w_{2} \left( {x,y,t} \right) = \frac{{5\sqrt[3]{{{\Omega }_{1}^{2} + {\Omega }_{2}^{2} }}}}{{8 6^{2/3} \sqrt[3]{\mu }{\Omega }_{3}^{2/3} }} - \frac{{\left( {\sqrt[3]{3}\sqrt[3]{{{\Omega }_{1}^{2} + {\Omega }_{2}^{2} }}\sqrt[3]{{{\Omega }_{3} }}} \right)}}{{2^{2/3} \sqrt[3]{\mu }}}\left( {\frac{1}{{24{\Omega }_{3} }} + \frac{{{\text{coth}}\left( {\frac{{\alpha_{1} x + \alpha_{2} y + \alpha_{3} t}}{{\sqrt { - 96{\Omega }_{3}^{2} } }}} \right)}}{{\sqrt { - 24{\Omega }_{3}^{2} } }}} \right),$$

9 $$\begin{gathered} w_{3} \left( {x,y,t} \right) = \frac{{5\sqrt[3]{{{\Omega }_{1}^{2} + {\Omega }_{2}^{2} }}}}{{8 6^{2/3} \sqrt[3]{\mu }{\Omega }_{3}^{2/3} }} - \frac{{\left( {\sqrt[3]{3}\sqrt[3]{{{\Omega }_{1}^{2} + {\Omega }_{2}^{2} }}\sqrt[3]{{{\Omega }_{3} }}} \right)}}{{2^{2/3} \sqrt[3]{\mu }}}\left( {\frac{1}{{24{\Omega }_{3} }} + \frac{{{\text{tanh}}\left( {\frac{{\alpha_{1} x + \alpha_{2} y + \alpha_{3} t}}{{\sqrt { - 24{\Omega }_{3}^{2} } }}} \right) + i{\text{sech}}\left( {\frac{{\alpha_{1} x + \alpha_{2} y + \alpha_{3} t}}{{\sqrt { - 24{\Omega }_{3}^{2} } }}} \right)}}{{\sqrt { - 24{\Omega }_{3}^{2} } }}} \right), \hfill \\ \hfill \\ \end{gathered}$$

10 $$w_{4} \left( {x,y,t} \right) = \frac{{5\sqrt[3]{{{\Omega }_{1}^{2} + {\Omega }_{2}^{2} }}}}{{8 6^{2/3} \sqrt[3]{\mu }{\Omega }_{3}^{2/3} }} - \frac{{\left( {\sqrt[3]{3}\sqrt[3]{{{\Omega }_{1}^{2} + {\Omega }_{2}^{2} }}\sqrt[3]{{{\Omega }_{3} }}} \right)}}{{2^{2/3} \sqrt[3]{\mu }}}\left( {\frac{1}{{24{\Omega }_{3} }} + \frac{{{\text{coth}}\left( {\frac{{\alpha_{1} x + \alpha_{2} y + \alpha_{3} t}}{{\sqrt { - 24{\Omega }_{3}^{2} } }}} \right) + {\text{csch}}\left( {\frac{{\alpha_{1} x + \alpha_{2} y + \alpha_{3} t}}{{\sqrt { - 24{\Omega }_{3}^{2} } }}} \right)}}{{\sqrt { - 24{\Omega }_{3}^{2} } }}} \right),$$

11 $$w_{5} \left( {x,y,t} \right) = \frac{{5\sqrt[3]{{{\Omega }_{1}^{2} + {\Omega }_{2}^{2} }}}}{{8 6^{2/3} \sqrt[3]{\mu }{\Omega }_{3}^{2/3} }} - \frac{{\left( {\sqrt[3]{3}\sqrt[3]{{{\Omega }_{1}^{2} + {\Omega }_{2}^{2} }}\sqrt[3]{{{\Omega }_{3} }}} \right)}}{{2 2^{2/3} \sqrt[3]{\mu }}}\left( {\frac{1}{{12{\Omega }_{3} }} + \frac{{{\text{tanh}}\left( {\frac{{\alpha_{1} x + \alpha_{2} y + \alpha_{3} t}}{{\sqrt { - 384{\Omega }_{3}^{2} } }}} \right) + {\text{coth}}\left( {\frac{{\alpha_{1} x + \alpha_{2} y + \alpha_{3} t}}{{\sqrt { - 384{\Omega }_{3}^{2} } }}} \right)}}{{\sqrt { - 24{\Omega }_{3}^{2} } }}} \right),$$

12 $$w_{6} \left( {x,y,t} \right) = \frac{{5\sqrt[3]{{{\Omega }_{1}^{2} + {\Omega }_{2}^{2} }}}}{{8 6^{2/3} \sqrt[3]{\mu }{\Omega }_{3}^{2/3} }} + \frac{{\sqrt[3]{3}\sqrt[3]{{{\Omega }_{1}^{2} + {\Omega }_{2}^{2} }}\sqrt[3]{{{\Omega }_{3} }}}}{{2^{2/3} \sqrt[3]{\mu }}}\left( { - \frac{1}{{24{\Omega }_{3} }} + \frac{{\left[ {\sqrt {m^{2} + n^{2} } - m{\text{cosh}}\left( {\frac{{\alpha_{1} x + \alpha_{2} y + \alpha_{3} t}}{{\sqrt { - 24{\Omega }_{3}^{2} } }}} \right)} \right]}}{{\sqrt { - 24{\Omega }_{3}^{2} } \left( {m{\text{sinh}}\left( {\frac{{\alpha_{1} x + \alpha_{2} y + \alpha_{3} t}}{{\sqrt { - 24{\Omega }_{3}^{2} } }}} \right) + n} \right)}}} \right),$$

13 $$w_{7} \left( {x,y,t} \right) = \frac{{5\sqrt[3]{{{\Omega }_{1}^{2} + {\Omega }_{2}^{2} }}}}{{8 6^{2/3} \sqrt[3]{\mu }{\Omega }_{3}^{2/3} }} + \frac{{\sqrt[3]{3}\sqrt[3]{{{\Omega }_{1}^{2} + {\Omega }_{2}^{2} }}\sqrt[3]{{{\Omega }_{3} }}}}{{2^{2/3} \sqrt[3]{\mu }}}\left( { - \frac{1}{{24{\Omega }_{3} }} + \frac{{\left[ {\sqrt {m^{2} + n^{2} } + m{\text{cosh}}\left( {\frac{{\alpha_{1} x + \alpha_{2} y + \alpha_{3} t}}{{\sqrt { - 24{\Omega }_{3}^{2} } }}} \right)} \right]}}{{\sqrt { - 24{\Omega }_{3}^{2} } \left( {m{\text{sinh}}\left( {\frac{{\alpha_{1} x + \alpha_{2} y + \alpha_{3} t}}{{\sqrt { - 24{\Omega }_{3}^{2} } }}} \right) + n} \right)}}} \right).$$

where $m$ and $n$ are two non-zero real constants and satisfies $n^{2} - m^{2} > 0$,

14 $$w_{8} \left( {x,y,t} \right) = \frac{{5\sqrt[3]{{{\Omega }_{1}^{2} + {\Omega }_{2}^{2} }}}}{{8 6^{2/3} \sqrt[3]{\mu }{\Omega }_{3}^{2/3} }} + \frac{{25\sqrt[3]{{{\Omega }_{1}^{2} + {\Omega }_{2}^{2} }}\left( {192 6^{2/3} \sqrt[3]{\mu }{\Omega }_{3}^{5/3} } \right)^{ - 1} {\text{cosh}}\left( {\frac{{\alpha_{1} x + \alpha_{2} y + \alpha_{3} t}}{{\sqrt { - 96{\Omega }_{3}^{2} } }}} \right)}}{{\left( {\frac{{{\text{sinh}}\left( {\frac{{\alpha_{1} x + \alpha_{2} y + \alpha_{3} t}}{{\sqrt { - 96{\Omega }_{3}^{2} } }}} \right)}}{{\sqrt { - 24{\Omega }_{3}^{2} } }} - \frac{{{\text{cosh}}\left( {\frac{{\alpha_{1} x + \alpha_{2} y + \alpha_{3} t}}{{\sqrt { - 96{\Omega }_{3}^{2} } }}} \right)}}{{24{\Omega }_{3} }}} \right)}},$$

15 $$w_{9} \left( {x,y,t} \right) = \frac{{5\sqrt[3]{{{\Omega }_{1}^{2} + {\Omega }_{2}^{2} }}}}{{8 6^{2/3} \sqrt[3]{\mu }{\Omega }_{3}^{2/3} }} + \frac{{25\sqrt[3]{{{\Omega }_{1}^{2} + {\Omega }_{2}^{2} }}\left( {192 6^{2/3} \sqrt[3]{\mu }{\Omega }_{3}^{5/3} } \right)^{ - 1} {\text{sinh}}\left( {\frac{{\alpha_{1} x + \alpha_{2} y + \alpha_{3} t}}{{\sqrt { - 96{\Omega }_{3}^{2} } }}} \right)}}{{\left( {\frac{{{\text{sinh}}\left( {\frac{{\alpha_{1} x + \alpha_{2} y + \alpha_{3} t}}{{\sqrt { - 96{\Omega }_{3}^{2} } }}} \right)}}{{24{\Omega }_{3} }} - \frac{{{\text{cosh}}\left( {\frac{{\alpha_{1} x + \alpha_{2} y + \alpha_{3} t}}{{\sqrt { - 96{\Omega }_{3}^{2} } }}} \right)}}{{\sqrt { - 24{\Omega }_{3}^{2} } }}} \right)}},$$

16 $$w_{10} \left( {x,y,t} \right) = \frac{{5\sqrt[3]{{{\Omega }_{1}^{2} + {\Omega }_{2}^{2} }}}}{{8 6^{2/3} \sqrt[3]{\mu }{\Omega }_{3}^{2/3} }} + \frac{{25\sqrt[3]{{{\Omega }_{1}^{2} + {\Omega }_{2}^{2} }}\left( {192 6^{2/3} \sqrt[3]{\mu }{\Omega }_{3}^{5/3} } \right)^{ - 1} {\text{sinh}}\left( {\frac{{\alpha_{1} x + \alpha_{2} y + \alpha_{3} t}}{{\sqrt { - 96{\Omega }_{3}^{2} } }}} \right)}}{{\left( {\frac{{{\text{cosh}}\left( {\frac{{\alpha_{1} x + \alpha_{2} y + \alpha_{3} t}}{{\sqrt { - 96{\Omega }_{3}^{2} } }}} \right) \pm 1}}{{\sqrt { - 24{\Omega }_{3}^{2} } }} - \frac{{{\text{sinh}}\left( {\frac{{\alpha_{1} x + \alpha_{2} y + \alpha_{3} t}}{{\sqrt { - 96{\Omega }_{3}^{2} } }}} \right)}}{{24{\Omega }_{3} }}} \right)}},$$

17 $$w_{11} \left( {x,y,t} \right) = \frac{{5\sqrt[3]{{{\Omega }_{1}^{2} + {\Omega }_{2}^{2} }}}}{{8 6^{2/3} \sqrt[3]{\mu }{\Omega }_{3}^{2/3} }} + \frac{{25\sqrt[3]{{{\Omega }_{1}^{2} + {\Omega }_{2}^{2} }}\left( {192 6^{2/3} \sqrt[3]{\mu }{\Omega }_{3}^{5/3} } \right)^{ - 1} {\text{sinh}}\left( {\frac{{\alpha_{1} x + \alpha_{2} y + \alpha_{3} t}}{{\sqrt { - 24{\Omega }_{3}^{2} } }}} \right)}}{{\left( {\frac{{{\text{cosh}}\left( {\frac{{\alpha_{1} x + \alpha_{2} y + \alpha_{3} t}}{{\sqrt { - 24{\Omega }_{3}^{2} } }}} \right) + \frac{1}{{\sqrt { - 24{\Omega }_{3}^{2} } }}}}{{\sqrt { - 24{\Omega }_{3}^{2} } }} - \frac{{{\text{sinh}}\left( {\frac{{\alpha_{1} x + \alpha_{2} y + \alpha_{3} t}}{{\sqrt { - 24{\Omega }_{3}^{2} } }}} \right)}}{{24{\Omega }_{3} }}} \right)}},$$

18 $$w_{12} \left( {x,y,t} \right) = \frac{{5\sqrt[3]{{{\Omega }_{1}^{2} + {\Omega }_{2}^{2} }}}}{{8 6^{2/3} \sqrt[3]{\mu }{\Omega }_{3}^{2/3} }} + \frac{{25\sqrt[3]{{{\Omega }_{1}^{2} + {\Omega }_{2}^{2} }}\left( {192 6^{2/3} \sqrt[3]{\mu }{\Omega }_{3}^{5/3} } \right)^{ - 1} {\text{sinh}}\left( {\frac{{\alpha_{1} x + \alpha_{2} y + \alpha_{3} t}}{{\sqrt { - 24{\Omega }_{3}^{2} } }}} \right)}}{{\left( {\frac{{{\text{cosh}}\left( {\frac{{\alpha_{1} x + \alpha_{2} y + \alpha_{3} t}}{{\sqrt { - 24{\Omega }_{3}^{2} } }}} \right) \pm 1}}{{\sqrt { - 24{\Omega }_{3}^{2} } }} - \frac{{{\text{sinh}}\left( {\frac{{\alpha_{1} x + \alpha_{2} y + \alpha_{3} t}}{{\sqrt { - 24{\Omega }_{3}^{2} } }}} \right)}}{{24{\Omega }_{3} }}} \right)}}$$

**Case 2:** When ${b}_{2}^{2}-4{b}_{3}{b}_{1}<0$ and ${b}_{3}{b}_{1}\ne 0$, then the twelve type of trigonometric solutions exist such as,

19 $$w_{13} \left( {x,y,t} \right) = \frac{{5\sqrt[3]{{{\Omega }_{1}^{2} + {\Omega }_{2}^{2} }}}}{{8 6^{2/3} \sqrt[3]{\mu }{\Omega }_{3}^{2/3} }} + \frac{{\left( {\sqrt[3]{3}\sqrt[3]{{{\Omega }_{1}^{2} + {\Omega }_{2}^{2} }}\sqrt[3]{{{\Omega }_{3} }}} \right)}}{{2^{2/3} \sqrt[3]{\mu }}}\left( { - \frac{1}{{24{\Omega }_{3} }} + \frac{{{\text{tan}}\left( {\frac{{\alpha_{1} x + \alpha_{2} y + \alpha_{3} t}}{{\sqrt {96{\Omega }_{3}^{2} } }}} \right)}}{{\sqrt {24{\Omega }_{3}^{2} } }}} \right),$$

20 $$w_{14} \left( {x,y,t} \right) = \phi_{13} \left( {x,y,t} \right) = \frac{{5\sqrt[3]{{{\Omega }_{1}^{2} + {\Omega }_{2}^{2} }}}}{{8 6^{2/3} \sqrt[3]{\mu }{\Omega }_{3}^{2/3} }} + \frac{{\left( {\sqrt[3]{3}\sqrt[3]{{{\Omega }_{1}^{2} + {\Omega }_{2}^{2} }}\sqrt[3]{{{\Omega }_{3} }}} \right)}}{{2^{2/3} \sqrt[3]{\mu }}}\left( {\frac{1}{{24{\Omega }_{3} }} + \frac{{{\text{cot}}\left( {\frac{{\alpha_{1} x + \alpha_{2} y + \alpha_{3} t}}{{\sqrt {96{\Omega }_{3}^{2} } }}} \right)}}{{\sqrt {24{\Omega }_{3}^{2} } }}} \right),$$

21 $$w_{15} \left( {x,y,t} \right) = \frac{{5\sqrt[3]{{{\Omega }_{1}^{2} + {\Omega }_{2}^{2} }}}}{{8 6^{2/3} \sqrt[3]{\mu }{\Omega }_{3}^{2/3} }} + \frac{{\left( {\sqrt[3]{3}\sqrt[3]{{{\Omega }_{1}^{2} + {\Omega }_{2}^{2} }}\sqrt[3]{{{\Omega }_{3} }}} \right)}}{{2^{2/3} \sqrt[3]{\mu }}}\left( { - \frac{1}{{24{\Omega }_{3} }} + \frac{{{\text{tan}}\left( {\frac{{\alpha_{1} x + \alpha_{2} y + \alpha_{3} t}}{{\sqrt {24{\Omega }_{3}^{2} } }}} \right) - {\text{sec}}\left( {\frac{{\alpha_{1} x + \alpha_{2} y + \alpha_{3} t}}{{\sqrt {24{\Omega }_{3}^{2} } }}} \right)}}{{\sqrt {24{\Omega }_{3}^{2} } }}} \right),$$

22 $$w_{16} \left( {x,y,t} \right) = \frac{{5\sqrt[3]{{\Omega_{1}^{2} + \Omega_{2}^{2} }}}}{{8 6^{2/3} \sqrt[3]{\mu }\Omega_{3}^{2/3} }} + \frac{{\left( {\sqrt[3]{3}\sqrt[3]{{\Omega_{1}^{2} + \Omega_{2}^{2} }}\sqrt[3]{{\Omega_{3} }}} \right)}}{{2^{2/3} \sqrt[3]{\mu }}}\left( { - \frac{1}{{24\Omega_{3} }} + \frac{{{\text{tan}}\left( {\frac{{\alpha_{1} x + \alpha_{2} y + \alpha_{3} t}}{{\sqrt {24\Omega_{3}^{2} } }}} \right) - {\text{sec}}\left( {\frac{{\alpha_{1} x + \alpha_{2} y + \alpha_{3} t}}{{\sqrt {24\Omega_{3}^{2} } }}} \right)}}{{\sqrt {24\Omega_{3}^{2} } }}} \right),$$

23 $$w_{17} \left( {x,y,t} \right) = \frac{{5\sqrt[3]{{{\Omega }_{1}^{2} + {\Omega }_{2}^{2} }}}}{{8 6^{2/3} \sqrt[3]{\mu }{\Omega }_{3}^{2/3} }} + \frac{{\left( {\sqrt[3]{3}\sqrt[3]{{{\Omega }_{1}^{2} + {\Omega }_{2}^{2} }}\sqrt[3]{{{\Omega }_{3} }}} \right)}}{{2 2^{2/3} \sqrt[3]{\mu }}}\left( { - \frac{1}{{12{\Omega }_{3} }} + \frac{{{\text{tan}}\left( {\frac{{\alpha_{1} x + \alpha_{2} y + \alpha_{3} t}}{{\sqrt {384{\Omega }_{3}^{2} } }}} \right) - {\text{cot}}\left( {\frac{{\alpha_{1} x + \alpha_{2} y + \alpha_{3} t}}{{\sqrt {384{\Omega }_{3}^{2} } }}} \right)}}{{\sqrt {24{\Omega }_{3}^{2} } }}} \right)$$

24 $$w_{18} \left( {x,y,t} \right) = \frac{{5\sqrt[3]{{{\Omega }_{1}^{2} + {\Omega }_{2}^{2} }}}}{{8 6^{2/3} \sqrt[3]{\mu }{\Omega }_{3}^{2/3} }} + \frac{{\left( {\sqrt[3]{3}\sqrt[3]{{{\Omega }_{3} }}\sqrt[3]{{{\Omega }_{1}^{2} + {\Omega }_{2}^{2} }}} \right)}}{{2^{2/3} \sqrt[3]{\mu }}}\left( {\frac{{\frac{{\sqrt {n^{2} - m^{2} } }}{{\sqrt {24{\Omega }_{3}^{2} } }} - \frac{{m{\text{cos}}\left( {\frac{{\alpha_{3} t + \alpha_{1} x + \alpha_{2} y}}{{\sqrt {24{\Omega }_{3}^{2} } }}} \right)}}{{\sqrt {24{\Omega }_{3}^{2} } }}}}{{m{\text{sin}}\left( {\frac{{\alpha_{3} t + \alpha_{1} x + \alpha_{2} y}}{{\sqrt {24{\Omega }_{3}^{2} } }}} \right) + n}} - \frac{1}{{24{\Omega }_{3} }}} \right),$$

25 $$w_{19} \left( {x,y,t} \right) = \frac{{5\sqrt[3]{{{\Omega }_{1}^{2} + {\Omega }_{2}^{2} }}}}{{8 6^{2/3} \sqrt[3]{\mu }{\Omega }_{3}^{2/3} }} - \frac{{\left( {\sqrt[3]{3}\sqrt[3]{{{\Omega }_{1}^{2} + {\Omega }_{2}^{2} }}\sqrt[3]{{{\Omega }_{3} }}} \right)}}{{2 2^{2/3} \sqrt[3]{\mu }}}\left( {\frac{1}{{24{\Omega }_{3} }} + \frac{{{\text{cot}}\left( {\frac{{\alpha_{1} x + \alpha_{2} y + \alpha_{3} t}}{{\sqrt {24{\Omega }_{3}^{2} } }}} \right) - {\text{csc}}\left( {\frac{{\alpha_{1} x + \alpha_{2} y + \alpha_{3} t}}{{\sqrt {24{\Omega }_{3}^{2} } }}} \right)}}{{\sqrt {24{\Omega }_{3}^{2} } }}} \right),$$

where $m$ and $n$ are two non-zero real constants and satisfies $m^{2} - n^{2} > 0$,

26 $$w_{20} \left( {x,y,t} \right) = \frac{{5\sqrt[3]{{{\Omega }_{1}^{2} + {\Omega }_{2}^{2} }}}}{{8 6^{2/3} \sqrt[3]{\mu }{\Omega }_{3}^{2/3} }} - \frac{{25\sqrt[3]{{{\Omega }_{1}^{2} + {\Omega }_{2}^{2} }}\sqrt[3]{{{\Omega }_{1}^{2} + {\Omega }_{2}^{2} }}\left( {192 6^{2/3} \sqrt[3]{\mu }{\Omega }_{3}^{5/3} } \right)^{ - 1} {\text{cos}}\left( {\frac{{\alpha_{1} x + \alpha_{2} y + \alpha_{3} t}}{{\sqrt {96{\Omega }_{3}^{2} } }}} \right)}}{{\left( {\frac{{{\text{sin}}\left( {\frac{{\alpha_{1} x + \alpha_{2} y + \alpha_{3} t}}{{\sqrt {96{\Omega }_{3}^{2} } }}} \right)}}{{\sqrt {24{\Omega }_{3}^{2} } }} - \frac{{{\text{cos}}\left( {\frac{{\alpha_{1} x + \alpha_{2} y + \alpha_{3} t}}{{\sqrt {96{\Omega }_{3}^{2} } }}} \right)}}{{24{\Omega }_{3} }}} \right)}}.$$

27 $$w_{21} \left( {x,y} \right) = \frac{{5\sqrt[3]{{{\Omega }_{1}^{2} + {\Omega }_{2}^{2} }}}}{{8 6^{2/3} \sqrt[3]{\mu }{\Omega }_{3}^{2/3} }} + \frac{{25\sqrt[3]{{{\Omega }_{1}^{2} + {\Omega }_{2}^{2} }}\left( {192 6^{2/3} \sqrt[3]{\mu }{\Omega }_{3}^{5/3} } \right)^{ - 1} {\text{sin}}\left( {\frac{{\alpha_{1} x + \alpha_{2} y + \alpha_{3} t}}{{\sqrt {96{\Omega }_{3}^{2} } }}} \right)}}{{\left( {\frac{{\sqrt {\frac{1}{{{\Omega }_{3}^{2} }}} {\text{cos}}\left( {\frac{{\alpha_{1} x + \alpha_{2} y + \alpha_{3} t}}{{\sqrt {96{\Omega }_{3}^{2} } }}} \right)}}{{\sqrt {24{\Omega }_{3}^{2} } }} - \frac{{{\text{sin}}\left( {\frac{{\alpha_{1} x + \alpha_{2} y + \alpha_{3} t}}{{\sqrt {96{\Omega }_{3}^{2} } }}} \right)}}{{24{\Omega }_{3} }}} \right)}},$$

28 $$w_{22} \left( {x,y,t} \right) = \frac{{5\sqrt[3]{{{\Omega }_{1}^{2} + {\Omega }_{2}^{2} }}}}{{8 6^{2/3} \sqrt[3]{\mu }{\Omega }_{3}^{2/3} }} - \frac{{25\sqrt[3]{{{\Omega }_{1}^{2} + {\Omega }_{2}^{2} }}\left( {192 6^{2/3} \sqrt[3]{\mu }{\Omega }_{3}^{5/3} } \right)^{ - 1} {\text{cos}}\left( {\frac{{\alpha_{1} x + \alpha_{2} y + \alpha_{3} t}}{{\sqrt {24{\Omega }_{3}^{2} } }}} \right)}}{{\left( {\frac{{{\text{cos}}\left( {\frac{{\alpha_{1} x + \alpha_{2} y + \alpha_{3} t}}{{\sqrt {24{\Omega }_{3}^{2} } }}} \right)}}{{24{\Omega }_{3} }} + \frac{{{\text{sin}}\left( {\frac{{\alpha_{1} x + \alpha_{2} y + \alpha_{3} t}}{{\sqrt {24{\Omega }_{3}^{2} } }} \pm 1} \right)}}{{\sqrt {24{\Omega }_{3}^{2} } }}} \right)}},$$

29 $$w_{23} \left( {x,y,t} \right) = \frac{{5\sqrt[3]{{{\Omega }_{1}^{2} + {\Omega }_{2}^{2} }}}}{{8 6^{2/3} \sqrt[3]{\mu }{\Omega }_{3}^{2/3} }} + \frac{{25\sqrt[3]{{{\Omega }_{1}^{2} + {\Omega }_{2}^{2} }}(\left( {192 6^{2/3} \sqrt[3]{\mu }{\Omega }_{3}^{5/3} } \right))^{ - 1} {\text{sin}}\left( {\frac{{\alpha_{1} x + \alpha_{2} y + \alpha_{3} t}}{{\sqrt {24{\Omega }_{3}^{2} } }}} \right)}}{{\frac{{\left( {\left( {{\text{cos}}\left( {\frac{{\alpha_{1} x + \alpha_{2} y + \alpha_{3} t}}{{\sqrt {24{\Omega }_{3}^{2} } }}} \right) \pm 1} \right) - b_{3} {\text{sin}}\left( {\frac{{\alpha_{1} x + \alpha_{2} y + \alpha_{3} t}}{{\sqrt {24{\Omega }_{3}^{2} } }}} \right)} \right)}}{{\sqrt {24{\Omega }_{3}^{2} } }}}},$$

30 $$w_{24} \left( {x,y,t} \right) = \frac{{5\sqrt[3]{{{\Omega }_{1}^{2} + {\Omega }_{2}^{2} }}}}{{8 6^{2/3} \sqrt[3]{\mu }{\Omega }_{3}^{2/3} }} + \frac{{25\sqrt[3]{{{\Omega }_{1}^{2} + {\Omega }_{2}^{2} }}(\left( {96 6^{2/3} \sqrt[3]{\mu }{\Omega }_{3}^{5/3} } \right))^{ - 1} {\text{sin}}\left( {\frac{{\alpha_{1} x + \alpha_{2} y + \alpha_{3} t}}{{\sqrt {384{\Omega }_{3}^{2} } }}} \right){\text{cos}}\left( {\frac{{\alpha_{1} x + \alpha_{2} y + \alpha_{3} t}}{{\sqrt {384{\Omega }_{3}^{2} } }}} \right)}}{{\left( {\frac{{\sqrt {\frac{1}{{{\Omega }_{3}^{2} }}} {\text{cos}}^{2} \left( {\frac{{\alpha_{1} x + \alpha_{2} y + \alpha_{3} t}}{{\sqrt {384{\Omega }_{3}^{2} } }}} \right)}}{\sqrt 6 } - \frac{{{\text{sin}}\left( {\frac{{\alpha_{1} x + \alpha_{2} y + \alpha_{3} t}}{{\sqrt {384{\Omega }_{3}^{2} } }}} \right){\text{cos}}\left( {\frac{{\alpha_{1} x + \alpha_{2} y + \alpha_{3} t}}{{\sqrt {384{\Omega }_{3}^{2} } }}} \right)}}{{12{\Omega }_{3} }} - \frac{1}{{\sqrt {24{\Omega }_{3}^{2} } }}} \right)}}$$

**Case 3:** When $b_{1} = 0$ and $b_{2} b_{3} \ne 0$, then the two type of hyperbolic solutions exist such as,

31 $$w_{25} \left( {x,y,t} \right) = \frac{{5\sqrt[3]{{{\Omega }_{1}^{2} + {\Omega }_{2}^{2} }}}}{{8 6^{2/3} \sqrt[3]{\mu }{\Omega }_{3}^{2/3} }} - \frac{{d\sqrt[3]{{{\Omega }_{1}^{2} + {\Omega }_{2}^{2} }}}}{{4 6^{2/3} \sqrt[3]{\mu }{\Omega }_{3}^{2/3} \left( {d - {\text{sinh}}\left( {\frac{{\alpha_{1} x + \alpha_{2} y + \alpha_{3} t}}{{24{\Omega }_{3} }}} \right) + {\text{cosh}}\left( {\frac{{\alpha_{1} x + \alpha_{2} y + \alpha_{3} t}}{{24{\Omega }_{3} }}} \right)} \right)}}.$$

32 $$w_{26} \left( {x,y,t} \right) = \frac{{5\sqrt[3]{{{\Omega }_{1}^{2} + {\Omega }_{2}^{2} }}}}{{8 6^{2/3} \sqrt[3]{\mu }{\Omega }_{3}^{2/3} }} - \frac{{\sqrt[3]{{{\Omega }_{1}^{2} + {\Omega }_{2}^{2} }}\left( {{\text{sinh}}\left( {\frac{{\alpha_{1} x + \alpha_{2} y + \alpha_{3} t}}{{24{\Omega }_{3} }}} \right) + {\text{cosh}}\left( {\frac{{\alpha_{1} x + \alpha_{2} y + \alpha_{3} t}}{{24{\Omega }_{3} }}} \right)} \right)}}{{4 6^{2/3} \sqrt[3]{\mu }{\Omega }_{3}^{2/3} \left( {d + {\text{sinh}}\left( {\frac{{\alpha_{1} x + \alpha_{2} y + \alpha_{3} t}}{{24{\Omega }_{3} }}} \right) + {\text{cosh}}\left( {\frac{{\alpha_{1} x + \alpha_{2} y + \alpha_{3} t}}{{24{\Omega }_{3} }}} \right)} \right)}}.$$

**Case 4:** When $b_{1} = 0 = b_{2}$ and $b_{3} \ne 0$, then one type of rational solutions exist such as,

33 $$w_{27} \left( {x,y,t} \right) = \frac{{5\sqrt[3]{{{\Omega }_{1}^{2} + {\Omega }_{2}^{2} }}}}{{8 6^{2/3} \sqrt[3]{\mu }{\Omega }_{3}^{2/3} }} - \frac{{\sqrt[3]{6}b_{3} \sqrt[3]{{{\Omega }_{1}^{2} + {\Omega }_{2}^{2} }}\sqrt[3]{{{\Omega }_{3} }}}}{{\sqrt[3]{\mu }\left( {d_{1} + \frac{{\alpha_{1} x + \alpha_{2} y + \alpha_{3} t}}{{24{\Omega }_{3} }}} \right)}}.$$

In the next section we find the solution by the help of modified auxiliary equation method.

## Modified auxiliary equation technique

We take the solution of Eq. (3) and get the polynomials form as follows^45^,

34 $$\phi \left( \zeta \right) = \lambda_{0} + \mathop \sum \limits_{i = 1}^{M} \left[ {\lambda_{i} \omega^{{\left( {u\zeta } \right)i}} + \nu_{i} \omega^{{ - \left( {u\zeta } \right)i}} } \right],$$

where the constants $\lambda_{0}$, $\lambda_{i}$ and $\nu_{i}$ (i = 1,2,3,…M) that can be solved after that, here $\omega \left( \zeta \right)$ is simplify the solution that is given below.

35 $$u^{\prime}\left( \zeta \right) = \frac{1}{{{\text{ln}}\left( \omega \right)}}\left( {b_{1} + b_{2} \omega^{u\zeta } + b_{3} \omega^{ - u\zeta } } \right),$$

here, $b_{1}$, $b_{2}$, $b_{3}$ and $u$ with $u > 0$ $u \ne 1$ are arbitrary constant that are determine later. Homogenous balancing principle can be applied to find the value of $M$ in the previous Eq. (3) and we can enter $M = 1$ in Eq. (34)

36 $$\phi \left( \zeta \right) = \lambda_{0} + \lambda_{1} \omega^{u\zeta } + \nu_{1} \omega^{ - u\zeta }$$

Determine the derivatives of Eq. (36) by applying the Eq. (35) and replace in the Eq. (3). After simplifying, collecting the coefficients of the same power of $\omega^{{\left( {u\zeta } \right)j}}$ and $\omega^{{ - \left( {u\zeta } \right)j}}$ and set them then equal to zero in all polynomials to gain a system of equations. Apply mathematica to deal with the system of calculation and gain the family of solution as,

### Family of solutions

37 $$\lambda_{0} = \frac{{11\sqrt[3]{3}\sqrt[3]{{\zeta_{1}^{2} + \zeta_{2}^{2} }}}}{{112 2^{2/3} \zeta_{3}^{2/3} \sqrt[3]{\mu }}},\lambda_{1} = 0,\nu_{1} = - \frac{{\sqrt[3]{6}b_{2} \sqrt[3]{{\zeta_{1}^{2} + \zeta_{2}^{2} }}\sqrt[3]{{\zeta_{3} }}}}{{\sqrt[3]{\mu }}},b_{1} = \frac{23}{{336\zeta_{3} }},b_{3} = \frac{14641}{{451584b_{2} \zeta_{3}^{2} }}.$$

**Case-1:** When $b_{1}^{2} - 4b_{2} b_{3} < 0$ and $b_{3} \ne 0$, then the trigonometric solutions are exist such as,

38 $$w_{28} \left( {x,y,t} \right) = \frac{{11\sqrt[3]{3}\sqrt[3]{{\zeta_{1}^{2} + \zeta_{2}^{2} }}}}{{112 2^{2/3} \zeta_{3}^{2/3} \sqrt[3]{\mu }}} + \frac{{14641\sqrt[3]{{\zeta_{1}^{2} + \zeta_{2}^{2} }}}}{{37632 6^{2/3} \zeta_{3}^{5/3} \sqrt[3]{\mu }\left( {\frac{23}{{336\zeta_{3} }} + \frac{{{\text{tan}}\left( {\frac{{\zeta_{3} t + \zeta_{1} x + \zeta_{2} y}}{{\sqrt { - 32\zeta_{3}^{2} } }}} \right)}}{{\sqrt { - 8\zeta_{3}^{2} } }}} \right)}},$$

and

39 $$w_{29} \left( {x,y,t} \right) = w_{28} \left( {x,y,t} \right) = \frac{{11\sqrt[3]{3}\sqrt[3]{{\zeta_{1}^{2} + \zeta_{2}^{2} }}}}{{112 2^{2/3} \zeta_{3}^{2/3} \sqrt[3]{\mu }}} + \frac{{14641\sqrt[3]{{\zeta_{1}^{2} + \zeta_{2}^{2} }}}}{{37632 6^{2/3} \zeta_{3}^{5/3} \sqrt[3]{\mu }\left( {\frac{23}{{336\zeta_{3} }} + \frac{{{\text{cot}}\left( {\frac{{\zeta_{3} t + \zeta_{1} x + \zeta_{2} y}}{{\sqrt { - 32\zeta_{3}^{2} } }}} \right)}}{{\sqrt { - 8\zeta_{3}^{2} } }}} \right)}}.$$

**Case-2:** When $b_{1}^{2} - 4b_{2} b_{3} > 0$ and $b_{3} \ne 0$, then the hyperbolic solutions are exist such as,

40 $$w_{30} \left( {x,y,t} \right) = \frac{{11\sqrt[3]{3}\sqrt[3]{{\zeta_{1}^{2} + \zeta_{2}^{2} }}}}{{112 2^{2/3} \zeta_{3}^{2/3} \sqrt[3]{\mu }}} + \frac{{14641\sqrt[3]{{\zeta_{1}^{2} + \zeta_{2}^{2} }}}}{{37632 6^{2/3} \zeta_{3}^{5/3} \sqrt[3]{\mu }\left( {\frac{23}{{336\zeta_{3} }} + \frac{{{\text{tanh}}\left( {\frac{{\sqrt { - 32\zeta_{3}^{2} } \left( {\zeta_{3} t + \zeta_{1} x + \zeta_{2} y} \right)}}{{4\sqrt { - 32\zeta_{3}^{2} } }}} \right)}}{{\sqrt { - 8\zeta_{3}^{2} } }}} \right)}},$$

and

41 $$\phi_{31} \left( {x,y,t} \right) = \frac{{11\sqrt[3]{3}\sqrt[3]{{\zeta_{1}^{2} + \zeta_{2}^{2} }}}}{{112 2^{2/3} \zeta_{3}^{2/3} \sqrt[3]{\mu }}} + \frac{{14641\sqrt[3]{{\zeta_{1}^{2} + \zeta_{2}^{2} }}}}{{37632 6^{2/3} \zeta_{3}^{5/3} \sqrt[3]{\mu }\left( {\frac{23}{{336\zeta_{3} }} + \frac{{{\text{tanh}}\left( {\frac{{\sqrt { - 32\zeta_{3}^{2} } \left( {\zeta_{3} t + \zeta_{1} x + \zeta_{2} y} \right)}}{{4\sqrt { - 32\zeta_{3}^{2} } }}} \right)}}{{\sqrt { - 8\zeta_{3}^{2} } }}} \right)}}.$$

**Case-3:** When $b_{1}^{2} - 4b_{2} b_{3} = 0$ and $b_{3} \ne 0$, then the rational solutions are exist such as,

42 $$w_{32} \left( {x,y,t} \right) = \frac{{11\sqrt[3]{3}\sqrt[3]{{\zeta_{1}^{2} + \zeta_{2}^{2} }}}}{{112 2^{2/3} \zeta_{3}^{2/3} \sqrt[3]{\mu }}} + \frac{{14641\sqrt[3]{{\zeta_{1}^{2} + \zeta_{2}^{2} }}\left( {\zeta_{3} t + \zeta_{1} x + \zeta_{2} y} \right)}}{{37632 6^{2/3} \zeta_{3}^{5/3} \sqrt[3]{\mu }\left( {\frac{{23\left( {\zeta_{3} t + \zeta_{1} x + \zeta_{2} y} \right)}}{{336\zeta_{3} }} + 2} \right)}}.$$

## Stability

This part examined at the sobolev type equations stability analysis. We determined the transformation as,

43 $$R = S + \varphi w\left( {x,y,t} \right),$$

Apply this value, we gain $S$ is the constant and steady state solution of Eq. (1),

44 $$- \mu S^{3} - 3\mu S^{2} \varphi w - 3\mu S\varphi^{2} w^{2} + \varphi w_{{{\text{xx}}}} + \varphi w_{{{\text{xxt}}}} + \varphi w_{{{\text{yy}}}} + \varepsilon w_{{{\text{yyt}}}} - \mu \varphi^{3} w^{3} = 0,$$

now, Linearising in the form $\varepsilon$, we get

45 $$- 3\mu S^{2} \varphi w + \varphi w_{{{\text{xx}}}} + \varphi w_{{{\text{xxt}}}} + \varphi w_{{{\text{yy}}}} + \varphi w_{{{\text{yyt}}}} = 0,$$

Assume the following solution to the previous equation,

46 $$w\left( {x,y,t} \right) = \gamma_{1} e^{{i{\Upsilon }_{1} x + i{\Upsilon }_{2} y + i{\Omega }t}} ,$$

where ${\Upsilon }_{1}$, ${\Upsilon }_{2}$ and ${\Omega }$ are the normalized wave number and frequency. Replace this transformation in Eq. (45),

47 $$- \gamma_{1} {\Upsilon }_{1}^{2} i{\Omega }\varphi - \gamma_{1} {\Upsilon }_{2}^{2} i{\Omega }\varphi - \gamma_{1} {\Upsilon }_{1}^{2} \varphi - \gamma_{1} {\Upsilon }_{2}^{2} \varphi - 3\gamma_{1} \mu S^{2} \varphi = 0,$$

after solving the previous equation we gain the value of ${\Omega }$ as,

48 $${\Omega } = i\left( {1 - \frac{{3\mu S^{2} }}{{{\Upsilon }_{1}^{2} + {\Upsilon }_{2}^{2} }}} \right).$$

Since the value of the ${\Omega }$ is imaginary, there will be an exponential growth in perturbation and no apparent decay in the superposition of the solution. This indicates an unstable dispersion.

## Physical representation

**Figure 1:**
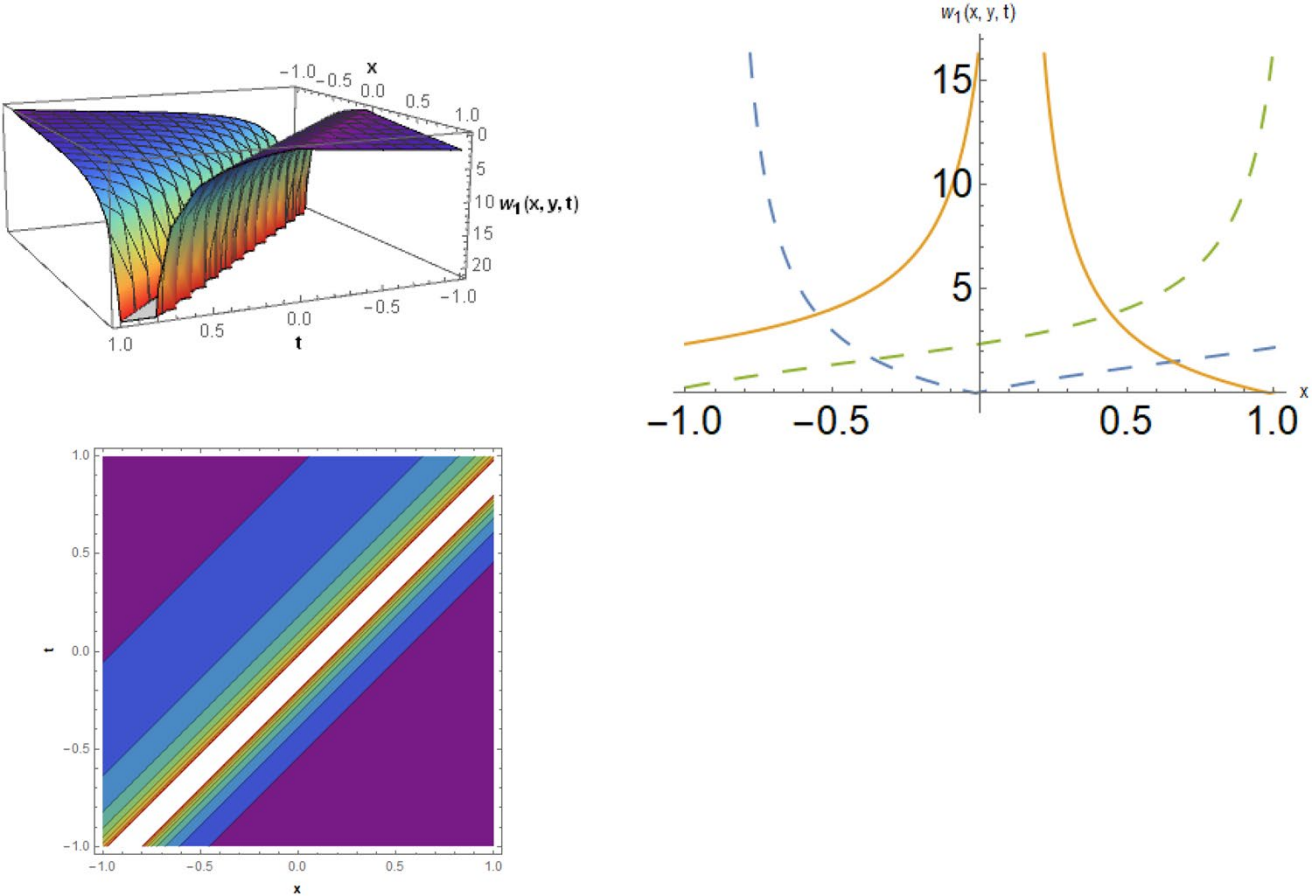
The dark soliton behavior for the solution $w_{1} \left( {x,y,t} \right)$ using $\alpha_{1} = 0.1,\alpha_{2} = 0.3,\alpha_{3} = - 0.1,\mu = 0.5,y = 1.5,{\Omega }_{1} = 0.2,{\Omega }_{2} = 0.1,{\Omega }_{3} = 0.01.$

**Figure 2:**
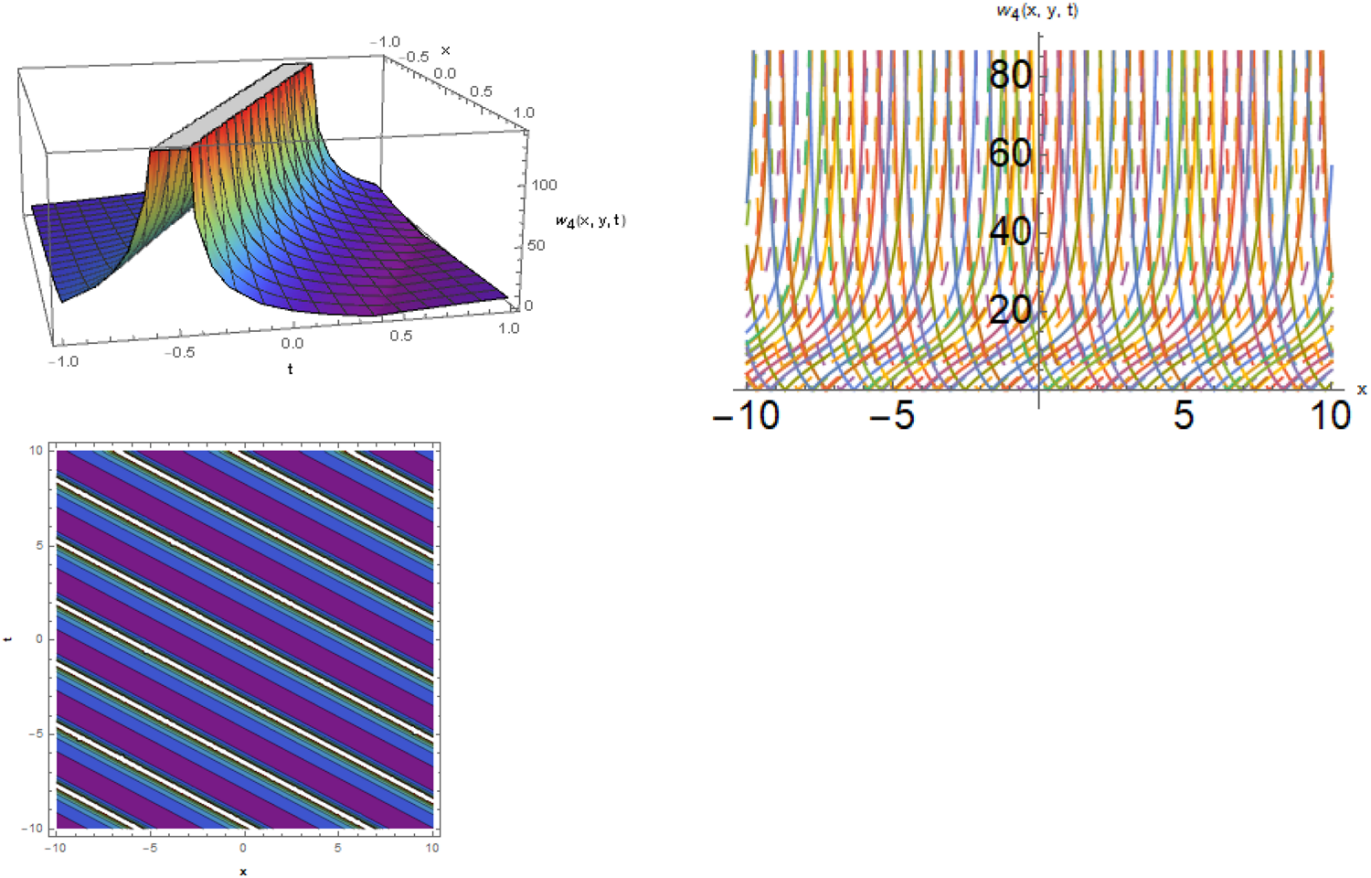
The bright soliton behavior for the solution $w_{4} \left( {x,y,t} \right)$ using $\alpha_{1} = 0.1,\alpha_{2} = 0.3,\alpha_{3} = - 0.1,\mu = 0.5,y = 1.5,{\Omega }_{1} = 0.2,{\Omega }_{2} = 3.9,{\Omega }_{3} = 0.01.$

**Figure 3:**
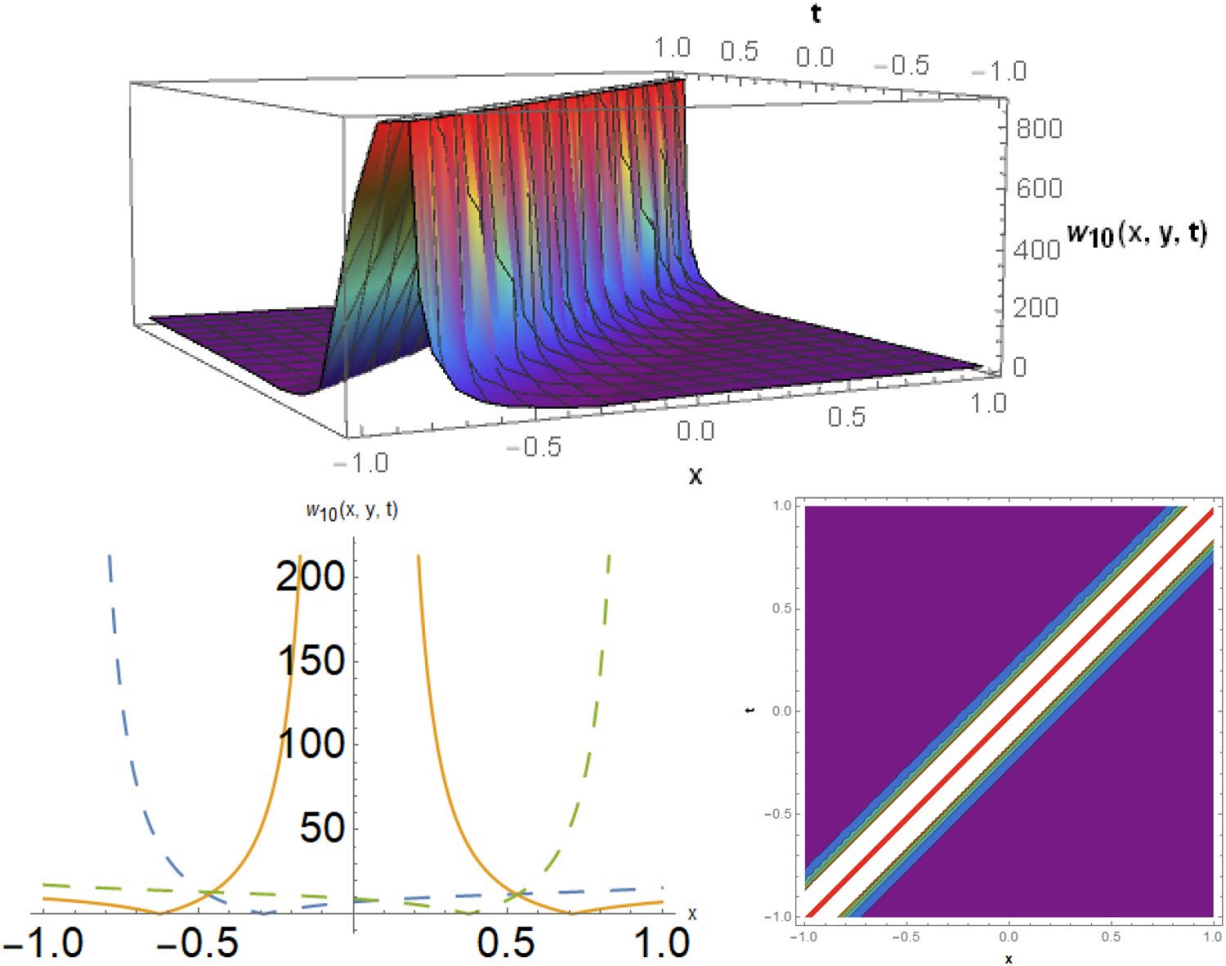
The bright soliton behavior for the solution $w_{10} \left( {x,y,t} \right)$ using $\alpha_{1} = 0.1,\alpha_{2} = 0.3,\alpha_{3} = - 0.1,\mu = 0.5,y = 1.5,{\Omega }_{1} = 0.2,{\Omega }_{2} = 3.9,{\Omega }_{3} = 0.01.$

**Figure 4:**
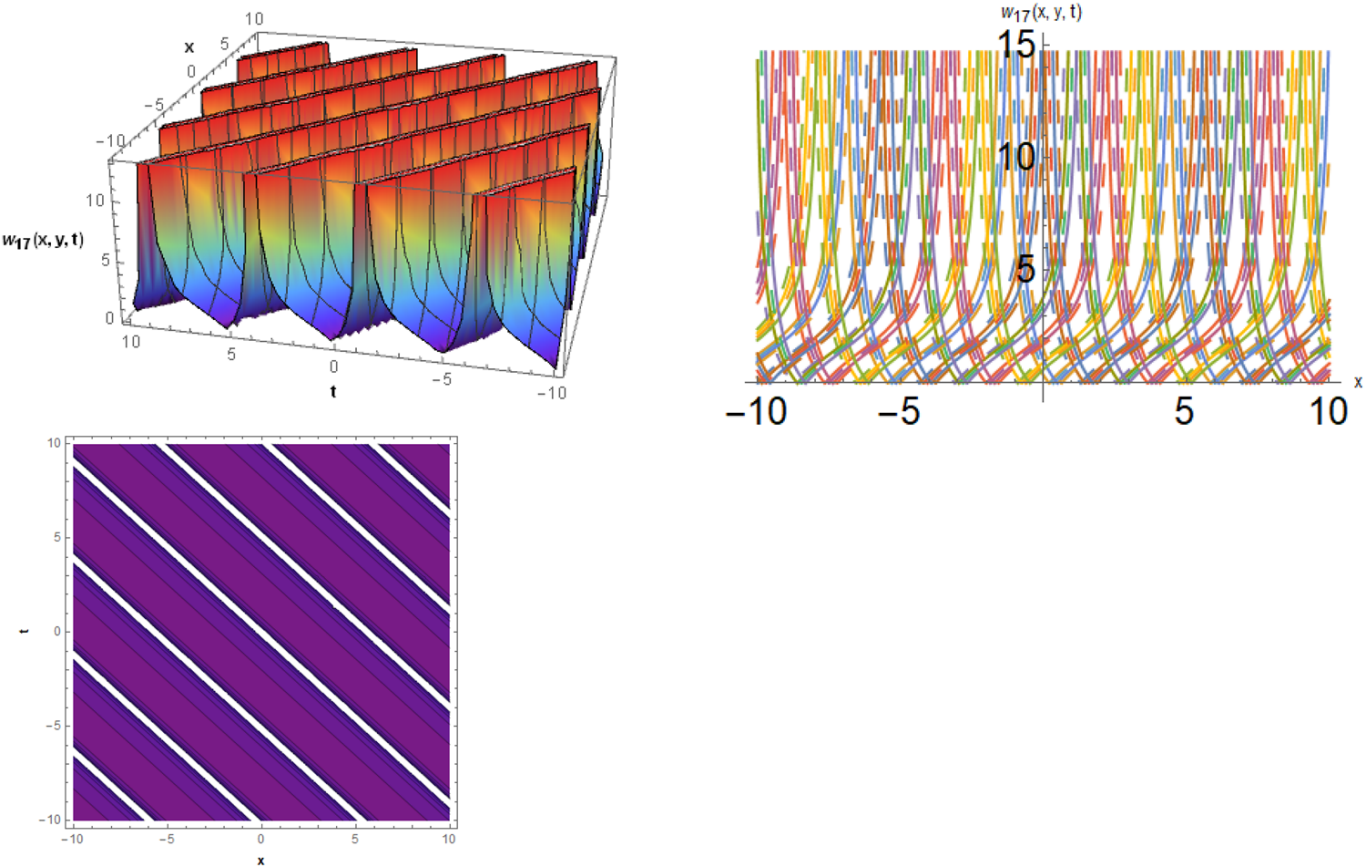
The solitary wave behavior for the solution $w_{17} \left( {x,y,t} \right)$ using $\alpha_{1} = 0.99,\alpha_{2} = 0.1,\alpha_{3} = 1.2,\mu = 0.2,y = 0.5,{\Omega }_{1} = 2.5,{\Omega }_{2} = 0.1,and{\Omega }_{3} = 0.2).$

**Figure 5:**
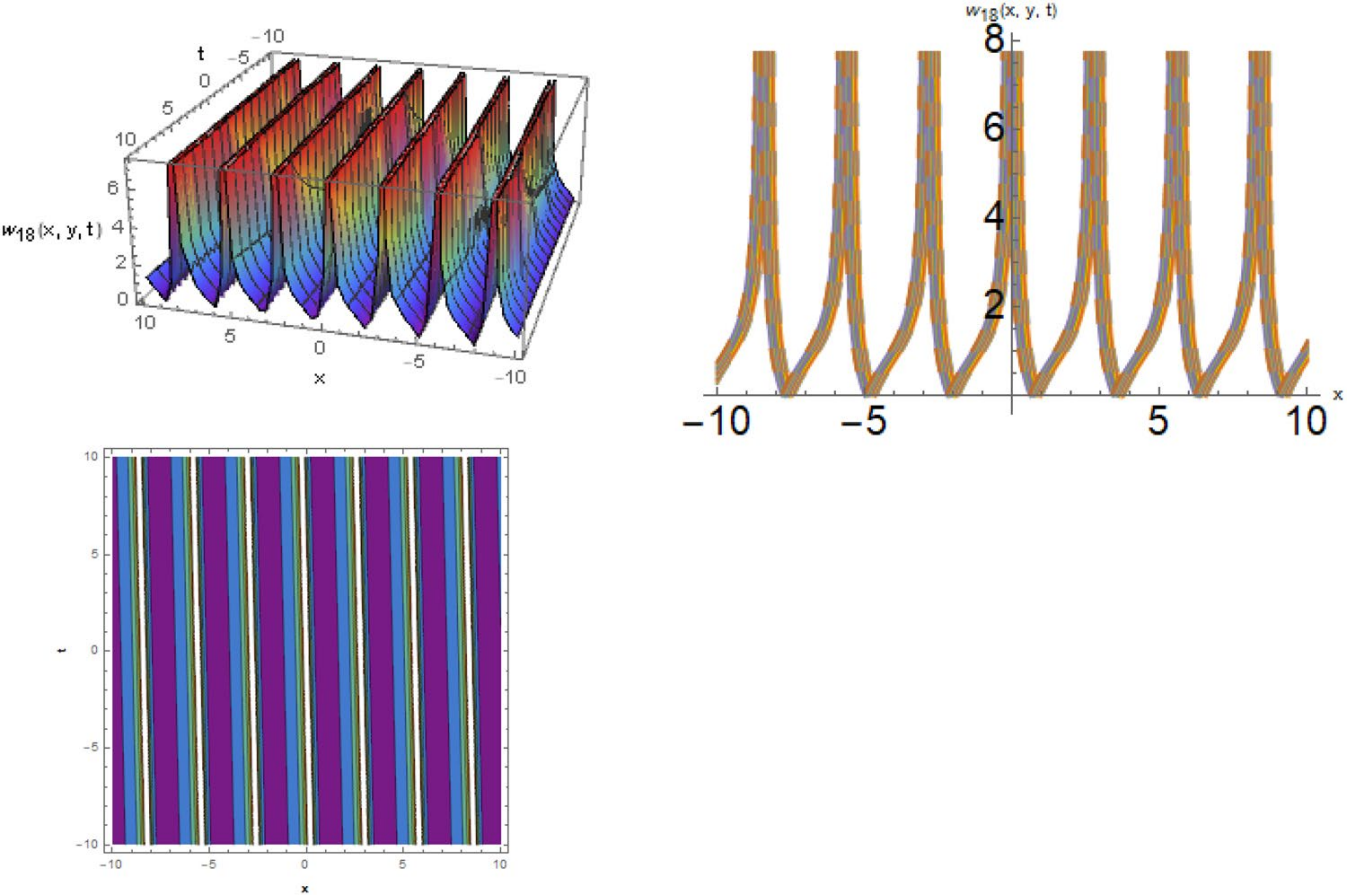
The solitary wave behavior for the solution $w_{18} \left( {x,y,t} \right)$ using $\alpha_{1} = 2.1,\alpha_{2} = 0.1,\alpha_{3} = 0.05,\mu = 0.3,y = 0.5,{\Omega }_{1} = 0.5,{\Omega }_{2} = 1.1,{\Omega }_{3} = 0.2.$

**Figure 6:**
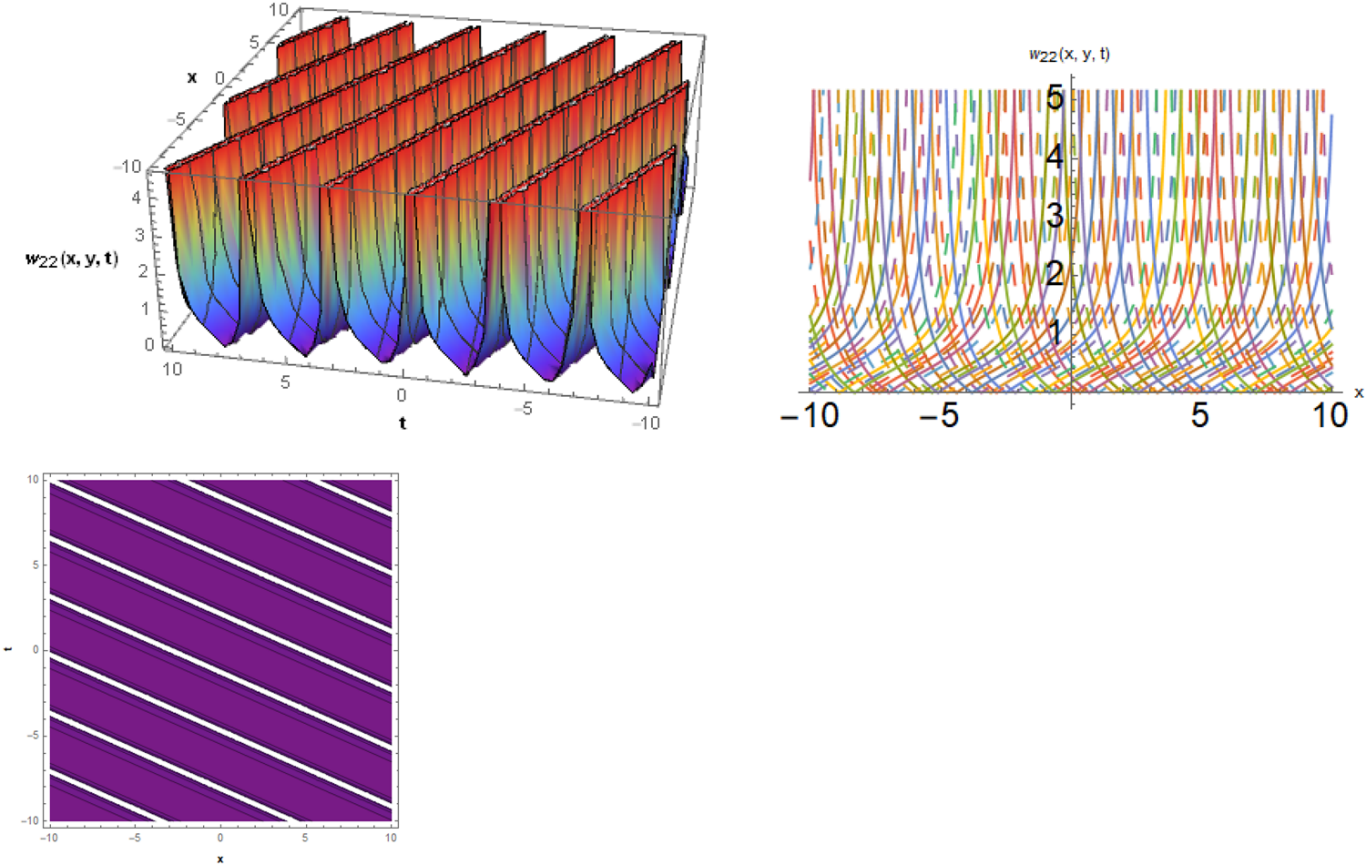
The solitary wave behavior for the solution $w_{22} \left( {x,y,t} \right)$ using $\alpha_{1} = 0.4,\alpha_{2} = 0.1,\alpha_{3} = 0.9,\mu = 0.3,y = 0.5,{\Omega }_{1} = 2.5,{\Omega }_{2} = 0.1,and{\Omega }_{3} = 0.2.$

**Figure 7:**
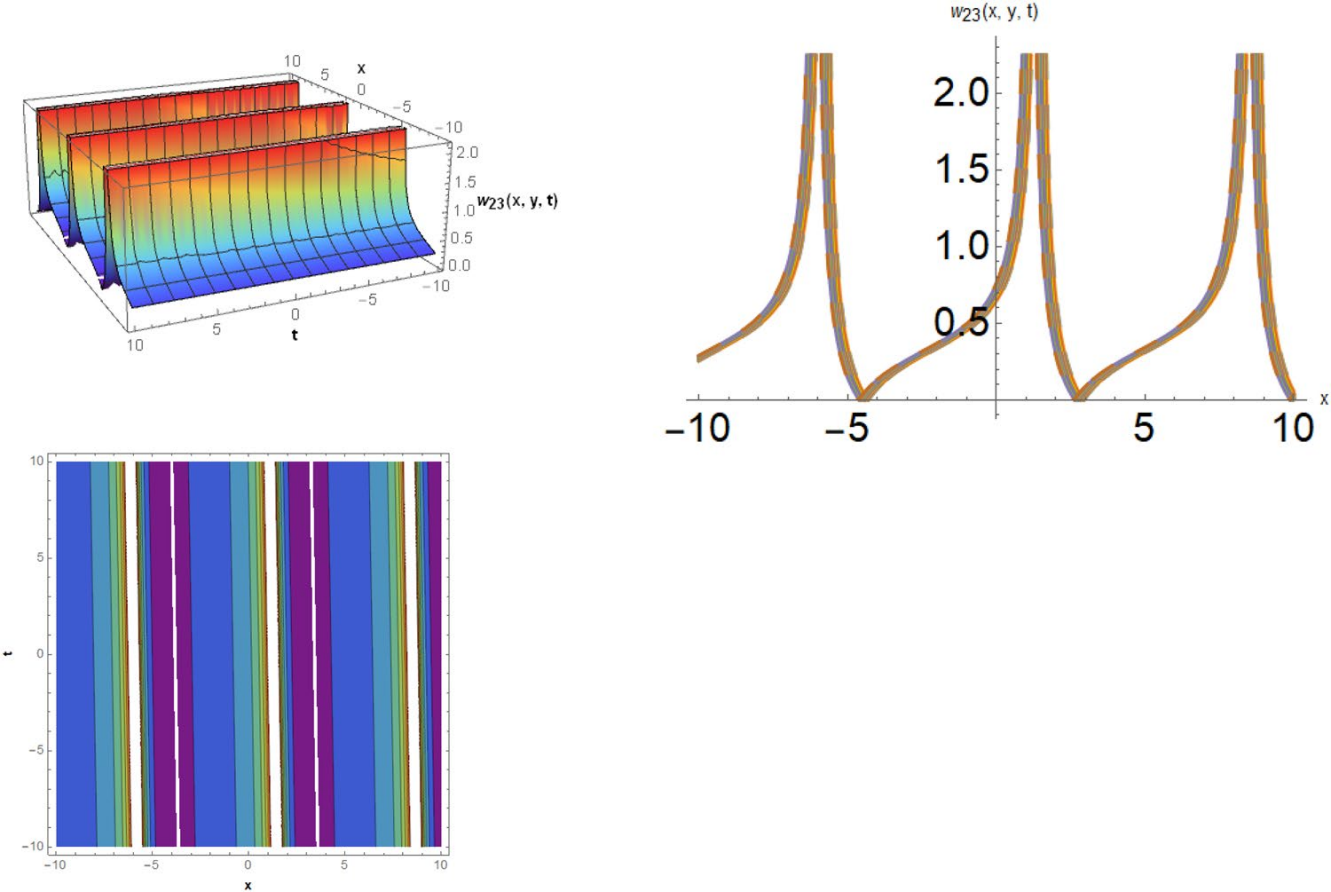
The solitary wave behavior for the solution $w_{23} \left( {x,y,t} \right)$ using $\alpha_{1} = 5.1,\alpha_{2} = 2.1,\alpha_{3} = 0.09,b_{3} = 1.3,\mu = 0.3,y = 0.5,{\Omega }_{1} = 2.5,{\Omega }_{2} = 3.1,{\Omega }_{3} = 1.2.$

**Figure 8:**
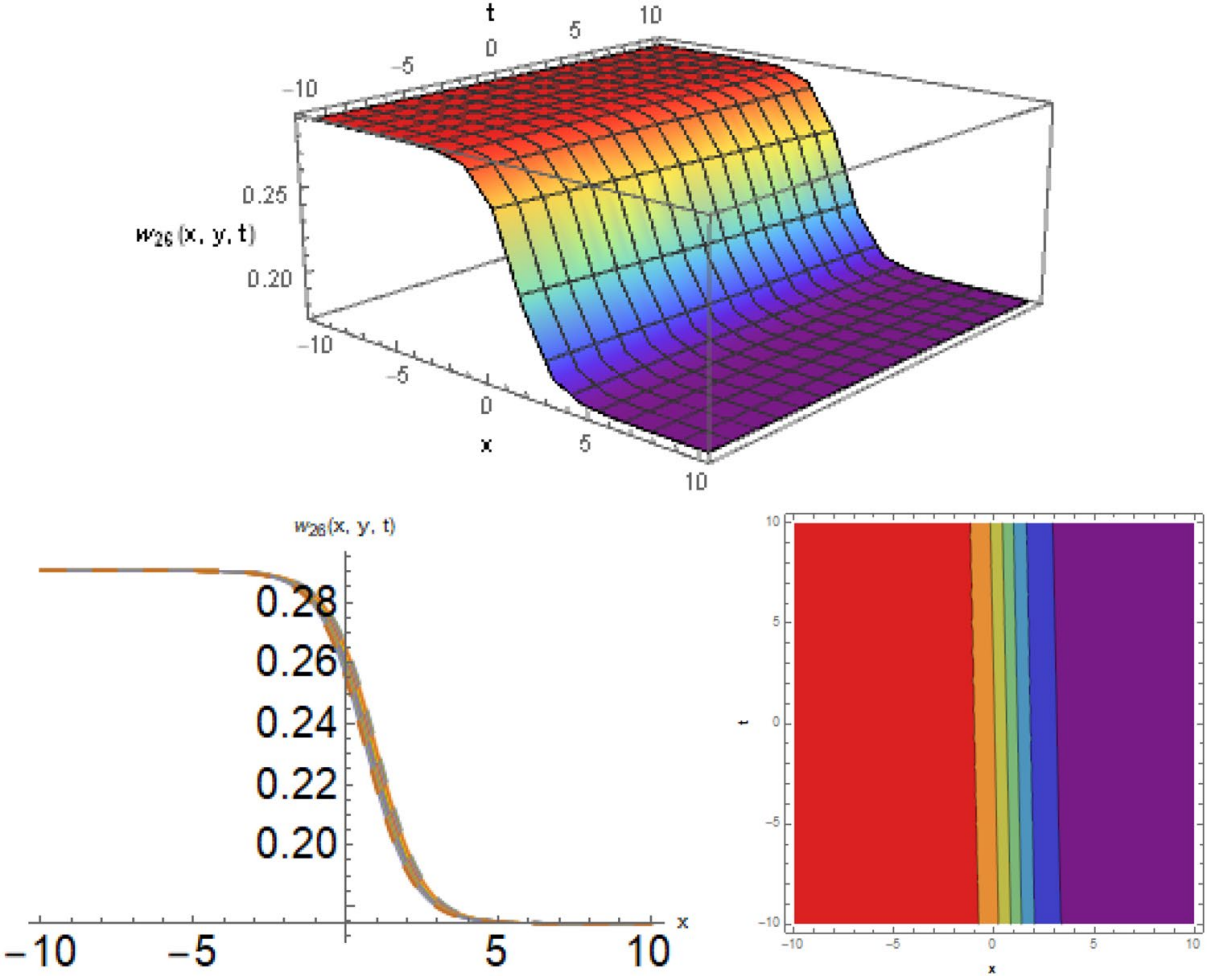
The kink soliton behavior for the solution for the solution $w_{26} \left( {x,y,t} \right)$ using $\alpha_{1} = 2.2,\alpha_{2} = 7.1,\alpha_{3} = 0.19,d = 2.9,\mu = 0.8,y = 1,{\Omega }_{1} = 0.5,{\Omega }_{2} = 0.1,and{\Omega }_{3} = 0.3.$

**Figure 9:**
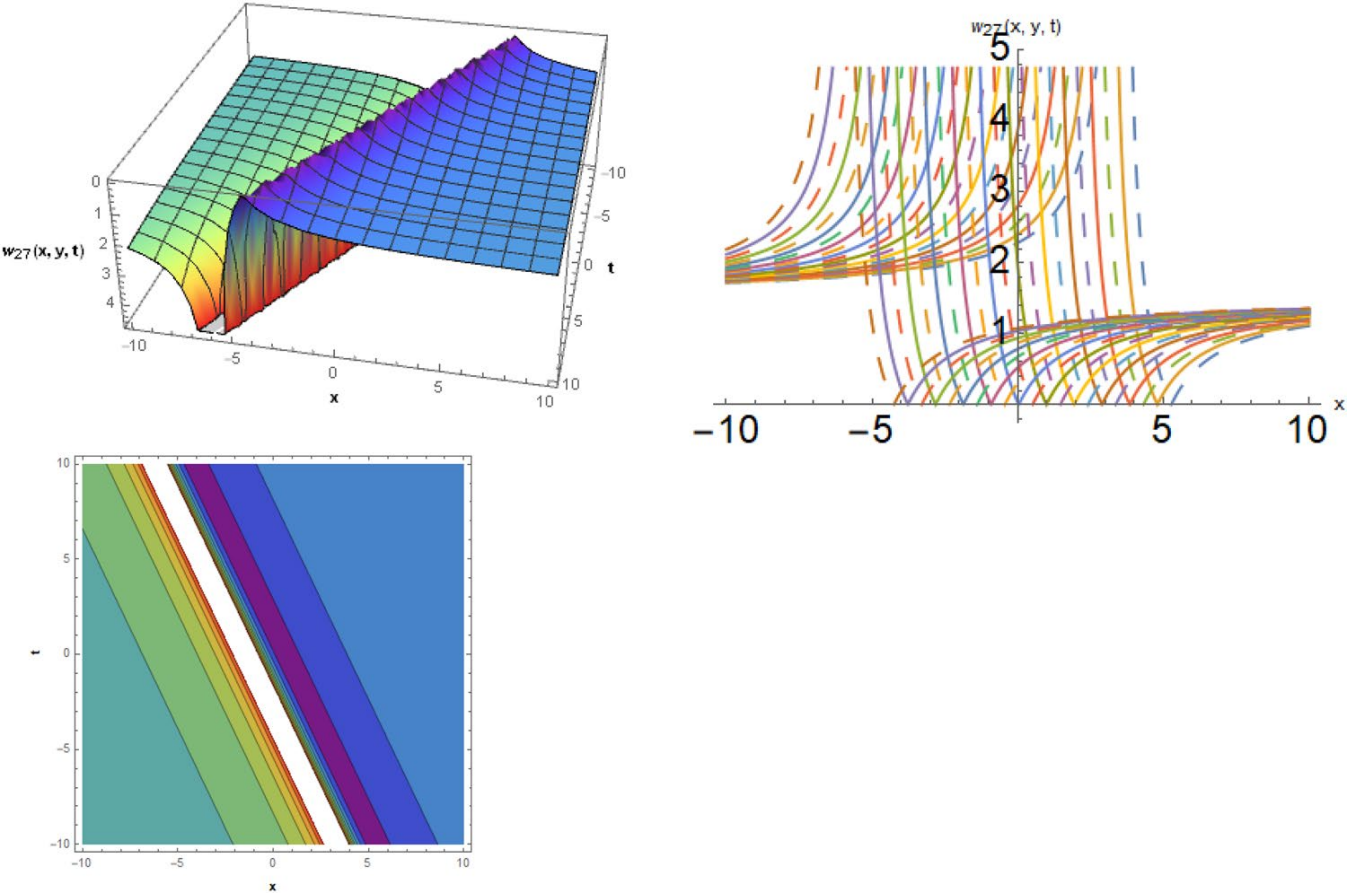
The dark-bright soliton behavior for the solution $w_{27} \left( {x,y,t} \right)$, using $\alpha_{1} = 2.1,\alpha_{2} = 0.4,\alpha_{3} = 1,b_{3} = 0.4,d_{1} = 0.5,\mu = 0.3,y = 0.5,{\Omega }_{1} = 2.5,{\Omega }_{2} = 0.1,and{\Omega }_{3} = 0.2.$

**Figure 10:**
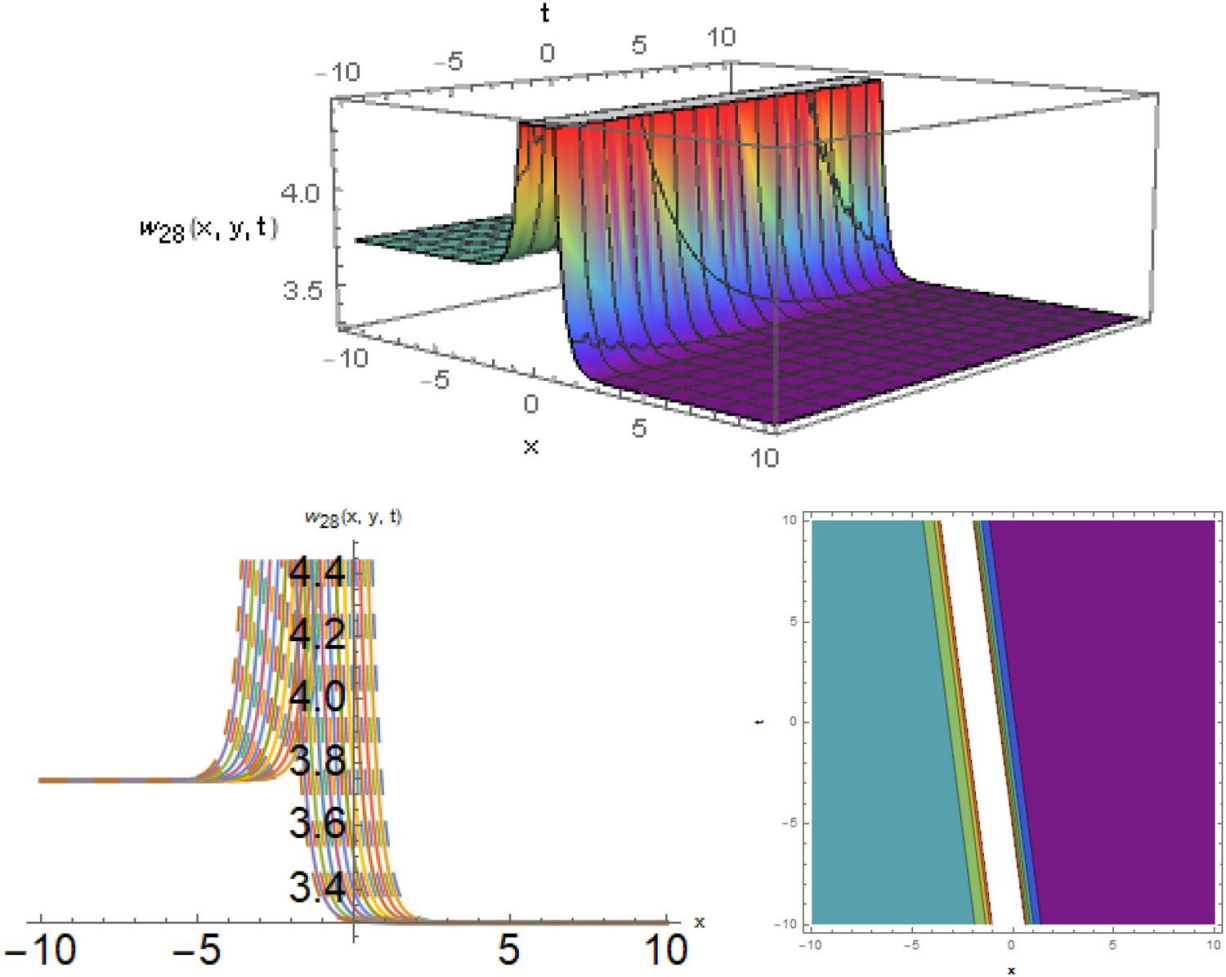
The singular soliton behavior for the solution $w_{28} \left( {x,y,t} \right)$, using $\zeta_{1} = 0.5,\zeta_{2} = 0.2,\zeta_{3} = 0.04,\mu = 3,andy = 1.$

**Figure 11:**
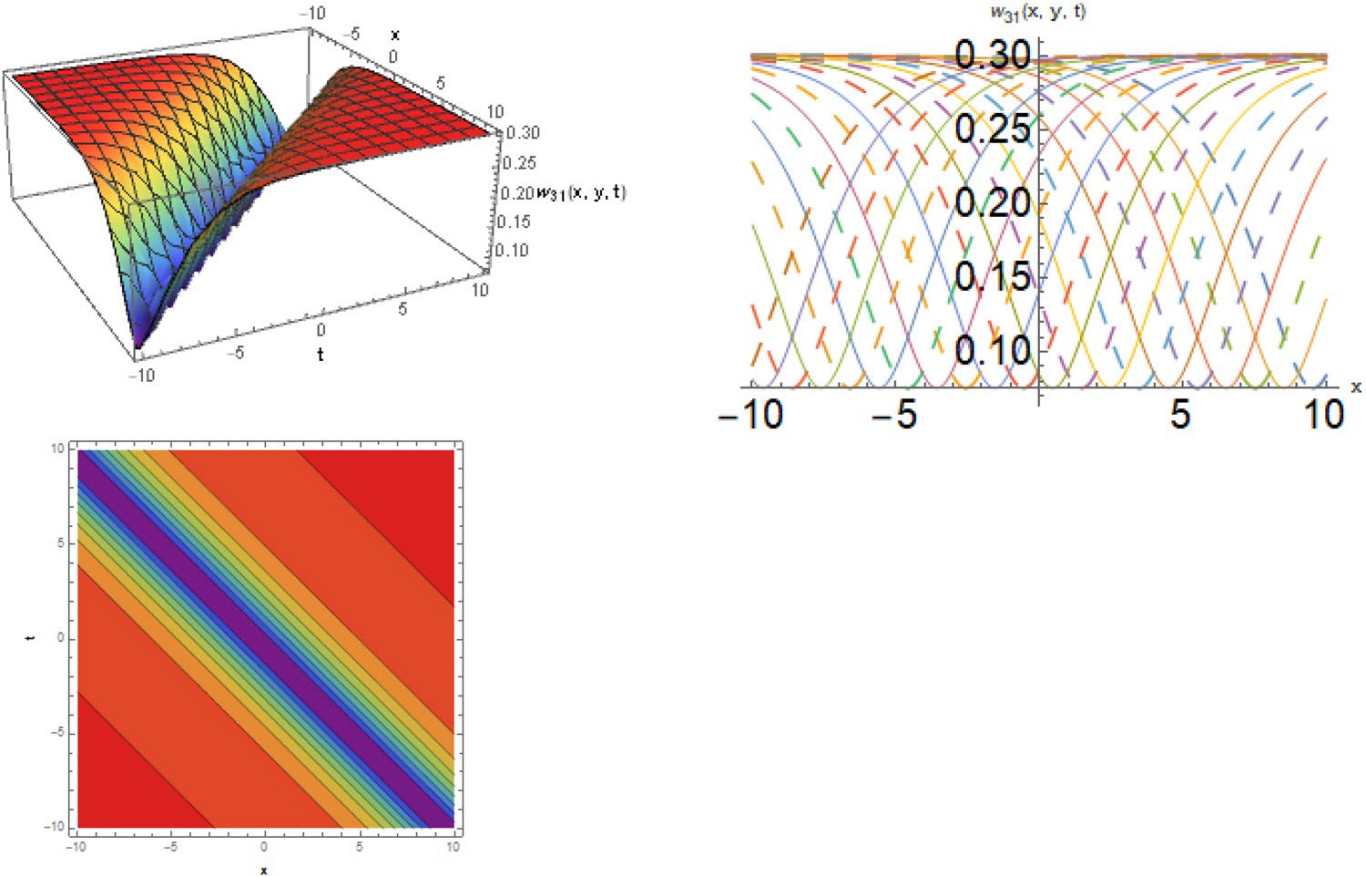
The dark soliton behavior for the solution $w_{31} \left( {x,y,t} \right)$, using $\zeta_{1} = 1.1,\zeta_{2} = 0.6,\zeta_{3} = 1.11,\mu = 2.1,\;and\;y = 1.$

**Figure 12:**
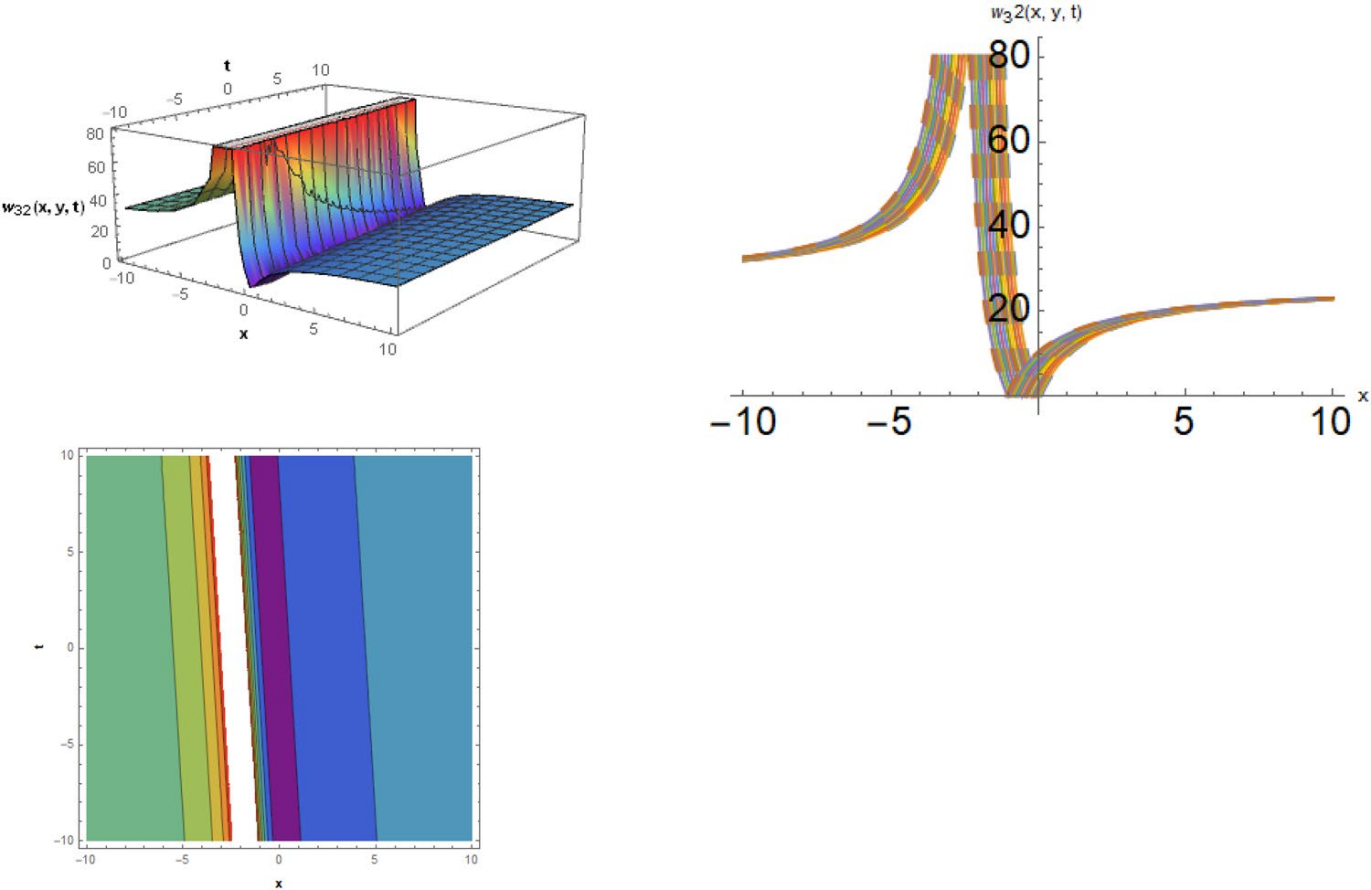
The dark-bright soliton behavior for the solution $w_{32} \left( {x,y,t} \right)$, using $\zeta_{1} = 0.5,\zeta_{2} = 0.2,\zeta_{3} = 0.03,\mu = 0.1,and\;y = 1.$

**Figure 13:**
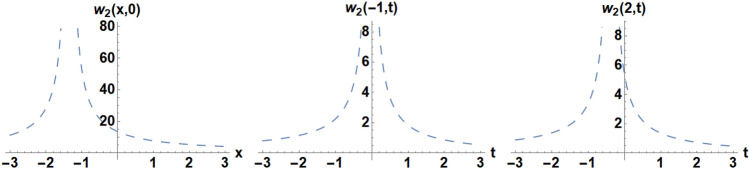
Diagrams of the initial and boundary values $w_{2} \left( {x,t} \right)$.

**Figure 14:**
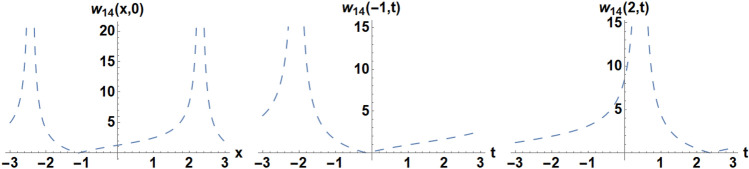
Diagrams of the initial and boundary values $w_{1} 4\left( {x,t} \right)$.

**Figure 15:**
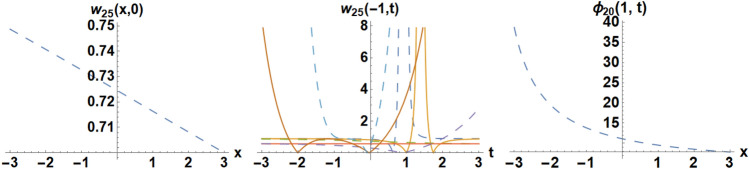
Diagrams of the initial and boundary values $w_{25} \left( {x,t} \right)$.

**Figure 16:**
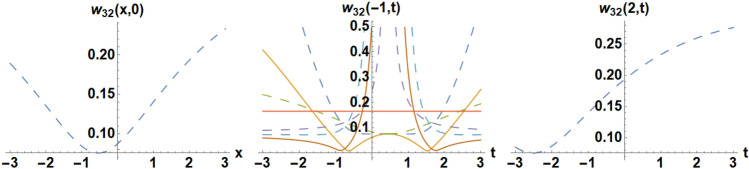
Diagrams of the initial and boundary values $w_{32} \left( {x,y,t} \right)$.

Here, examined is the graphical behavior of solutions given different parameter choices. Various families of solutions including singular periodic, periodic, singular wave, shock wave, shock-singular, periodic-singular, complex solitary-shock, and double singular have been efficiently gained. The gained results are very helpful in understanding the nonlinear dynamics for the comparison of experimental and numerical solutions. The 3D, line graphs and corresponding contours are drawn with the help of the $Mathematica$ software that explains the physical behavior of the state variable. The Figs. 1, 2, 3, 4, 5, 6, 7, 8, 9, 10, 11 are shows the dark soliton behavior while Figs. 2,3 are presents the bright soliton. Figures 4,5,6,7 are gives us the solitary wave representations. The kink soliton behavior is observed from the Fig. 8 while combined dark-bright behavior is shown in Figs. 9,12. The singular soliton behavior is drawn in the Fig. 10. These soliton behaviors are very fruitful for the current passing in the semiconductors that how the current travels from one place to another place. Diagrams of the initial and boundary values have been presented by Figs. 13, 14, 15, 16.

The physical explanation of our findings may be useful as a tool for future research into nonlinear wave problems in applied science.

## Selection of unique physical problem

In this section, we select the unique physical problems from the above solutions that help the researchers to take such problems for the sake of approximate solutions. Unique characteristics like as stability, non-dispersiveness, and the capacity to hold their form while travelling at constant speeds make exact soliton solutions to nonlinear partial differential equations intriguing solutions. In domains like nonlinear optics, plasma physics, and fluid dynamics, they are frequently employed to explain a variety of physical phenomena. Solitons behave differently in different circumstances, and their behaviour can be better understood by building unique physical problems with particular boundary conditions (BCs) and starting conditions (ICs) based on accurate soliton solutions.

### Example 1:

We pick the unique situations for solving Eq. (7) like,

the IC is selected as

49 $$w_{2} \left( {x,0} \right) = 0.12785 - 0.156583{\text{cot}}\left( {\left( {0.0850517 + 0.i} \right)\left( {0.1x + 0.135} \right)} \right),$$

the BCs are selected as,

50 $$w_{2} \left( { - 1,t} \right) = 0.12785 - 0.156583{\text{cot}}\left( {\left( {0.0850517 + 0.i} \right)\left( {0.9t + 0.035} \right)} \right),$$

51 $$w_{2} \left( {1,t} \right) = 0.12785 - 0.156583{\text{cot}}\left( {\left( {0.0850517 + 0.i} \right)\left( {0.9t + 0.335} \right)} \right).$$

### Example 2:

We pick the unique situations such as,

the IC is selected as

52 $$w_{14} \left( {x,0} \right) = 1.49295{\text{tan}}\left( {0.663403x + 0.0408248} \right) + 1.21899,$$

the BCs are selected as,

53 $$w_{14} \left( { - 1,t} \right) = 1.21899 - 1.49295{\text{tan}}\left( {0.622579 - 0.459279t} \right),$$

54 $$w_{14} \left( {1,t} \right) = 1.49295{\text{tan}}\left( {0.459279t + 1.36763} \right) + 1.21899.$$

### Example 3:

We pick the unique situations such as,

the IC is selected as

55 $$w_{25} \left( {x,0} \right) = 0.893855 - \frac{0.321788}{{ - 1.{\text{sinh}}\left( {0.0916667x} \right) + {\text{cosh}}\left( {0.0916667x} \right) + 0.9}},$$

the BCs are selected as,

56 $$w_{25} \left( { - 1,t} \right) = 0.893855 - \frac{0.293602}{{ - 1.{\text{sinh}}\left( {0.819444t} \right) + 1.{\text{cosh}}\left( {0.819444t} \right) + 0.821168}},$$

57 $$w_{25} \left( {1,t} \right) = 0.893855 - \frac{0.386536}{{ - 1.{\text{sinh}}\left( {0.819444t} \right) + 1.{\text{cosh}}\left( {0.819444t} \right) + 1.08109}}.$$

### Example 4:

We pick the unique situations for solving Eq. (38) such as,

the IC is selected as

58 $$\phi_{38} \left( {x,0} \right) = 0.0755451 + \frac{0.0898672}{{0.0616688 - \left( {0. + 0.318517i} \right){\text{coth}}\left( {\left( {0.25 + 0.i} \right)\left( {1.1x + 0.6} \right)} \right)}},$$

the BCs are selected as,

59 $$\phi_{38} \left( { - 1,t} \right) = 0.0755451 + \frac{0.0898672}{{0.0616688 - \left( {0. + 0.318517i} \right){\text{coth}}\left( {\left( {0.25 + 0.i} \right)\left( {1.11t - 0.5} \right)} \right)}},$$

60 $$\phi_{38} \left( {1,t} \right) = 0.0755451 + \frac{0.0898672}{{0.0616688 - \left( {0. + 0.318517i} \right){\text{coth}}\left( {\left( {0.25 + 0.i} \right)\left( {1.11t + 2.8} \right)} \right)}}.$$

## Conclusions

In this study, the Sobolev-type equation is considered analytically to explored the exact solitary wave solutions. These types of equation have their own importance in applied sciences due to the involvement of the mixed third order derivative. The Sobolev-type equations are found in a broad range of fields, such as ecology, fluid dynamics, soil mechanics, and thermodynamics. To, obtained the explicit solitary wave solutions we apply the two novel techniques namely as; generalized Riccati equation mapping and modified auxiliary equation (MAE) methods. The different types of abundant families of solutions in the form of dark soliton, bright soliton, solitary wave solutions, mixed singular soliton, mixed dark-bright soliton, periodic wave, and mixed periodic solutions. Moreover, we also discussed the linear stability analysis for the underlying model. Also, the specific physical problems with specific boundary conditions (BCs) and initial conditions (ICs) are constructed that are based on exact soliton solutions that can be help us better understand how solitons behave in various situations. Thus, selecting distinct physical problems (ICs and BCS) are also constructed from a range of solutions. The unique physical problems are selected from numerious solutions that will help the researchers to check the nonlinear dynamics in an accurate way. The 3D, line graphs and corresponding contours are drawn with the help of the Mathematica software that explains the physical behavior of the state variable.

### Data availability

The datasets used and/or analysed during the current study available from the corresponding author on reasonable request.
